# Opioid use and the risk of cancer incidence and mortality: a systematic review

**DOI:** 10.1007/s10555-025-10268-0

**Published:** 2025-06-11

**Authors:** Shakti Shrestha, Holly Foot, Mahdi Sheikh, Marie-Odile Parat, Adam La Caze

**Affiliations:** 1 School of Pharmacy and Pharmaceutical Sciences, The University of Queensland, Dutton Park, QLD 4102 Australia; 2 Genomic Epidemiology Branch, International Agency for Research On Cancer (IARC-WHO), Lyon, France

**Keywords:** Opioid exposure, Cancer mortality, Cancer incidence, Cancer risk

## Abstract

There is abundant but discrepant scientific literature reporting an effect of opioids on the course of cancer. The International Agency for Research on Cancer monographs recently classified opium consumption as carcinogenic to humans in certain organs, raising concerns this may be due at least in part to the alkaloids opium contains (such as morphine and codeine). This systematic review investigated whether opioid exposure among cancer-free individuals is independently associated with the risk of future cancer incidence or cancer mortality. An electronic database search was conducted in PubMed, EMBASE, Web of Science, PsycINFO, International Pharmaceutical Abstracts, CINAHL and Scopus. Studies were included if they provided a statistical estimate of cancer mortality, cancer incidence, or cancer risk following opioid exposure. Study quality was assessed using the Newcastle–Ottawa Scale. Study characteristics and outcomes were extracted and analysed in a descriptive narrative synthesis. There were 27 studies that met the inclusion criteria, representing a total of 4,542,745 participants. Twelve of the 27 were rated as high quality according to the Newcastle–Ottawa Scale. The observed data is consistent with a small increase in the risk of cancer incidence or cancer mortality following opioid exposure, particularly in a subset of organs. There is, however, considerable uncertainty in the evidence given the substantial risk of bias in estimating the overall effect of opioid exposure on cancer outcomes in these studies. This review synthesises studies reporting cancer risk following opioid exposure and identifies the key methodological factors influencing ongoing uncertainty estimating the true effect. Rigorous epidemiological studies employing specific methods to minimize bias are warranted.

**Supplementary Information:**

The online version contains supplementary material available at 10.1007/s10555-025-10268-0.

## Introduction

Pain is a common symptom experienced by cancer patients [1]. Similarly, chronic pain affects a substantial proportion of the general population [2]. Opioids are widely used to manage both cancer-related and non-cancer related pain. However, there is considerable controversy on the long-term effects of opioids. Whether the use of opioids in cancer patients can affect cancer prognosis remains debated [3, 4]. Furthermore, concerns have been raised about whether opioid use in the general population may influence future cancer risk [5, 6].

Cell culture and animal model studies have demonstrated that opioids modulate tumour biology through both positive and negative direct effects on tumour growth and metastasis, and indirect effects on immunity, inflammation and angiogenesis [7–14]. Opioid exposure in the perioperative period of the cancer surgery has received particular attention, with several authors postulating that opioids may increase tumour recurrence or metastasis following cancer surgery [15–17]. While retrospective observational studies comparing regional anaesthesia (low opioid exposure) to general anaesthesia (high opioid exposure) have provided discrepant findings [18], prospective randomized studies assessing the effects of different approaches to anaesthesia and opioid exposure on cancer recurrence failed to demonstrate a benefit to opioid-sparing techniques [19]. Thus, there is uncertainty regarding the overall effect of opioid exposure on cancer progression [20, 21], the extent to which the effects of opioids on tumour biology occur in different contexts (*in vitro*, preclinical or clinical), and whether or not there are differences in effect with different opioids [22, 23].

Most work to date assessing the effect of opioid exposure on cancer outcomes has focused on either experimental models or clinical contexts in which cancer is present. If opioid exposure promotes tumour progression through effects on the immune system, angiogenesis, or other mechanisms, future cancer risk would be expected to increase in cancer-free populations exposed to opioids. Importantly, opium consumption was recently classified as carcinogenic to humans by the International Agency for Research on Cancer (IARC) monographs, with evidence that the risk of opium-associated cancer being organ specific [24–26]. This classification further raises concerns on pharmaceutical opioids that are either derived from opium or synthesized to mimic its alkaloids.

This systematic review seeks to address the question: Is opioid exposure among cancer-free individuals independently associated with the risk of future cancer incidence or cancer mortality?

## Materials and methods

This review was conducted in accordance with the Preferred Reporting Items for Systematic Reviews and Meta Analysis (PRISMA) statement [27]. The study protocol was registered in the Open Science Framework [28].

### Search strategy and information sources

The search strategy was developed using the PICO (population, intervention, comparator and outcome) model for two key populations of interest; non-cancer patients with pain and patients receiving opioid replacement treatment. The intervention/exposure for each population was any currently marketed opioid use. The primary outcomes of interest were cancer mortality and incidence of cancer. The key terms and their synonyms represented in each PICO were combined using appropriate Boolean logic (see Appendix 1) to conduct a comprehensive literature search in seven electronic databases (PubMed, EMBASE, Web of Science, PsycINFO, International Pharmaceutical Abstracts, CINAHL and Scopus) and the results combined. The search was conducted from the inception of the relevant database to 28 October 2024.

### Selection process and eligibility criteria

All the identified studies were exported into Covidence to manage the selection process, which followed guidance provided in the Cochrane Handbook for Systematic Reviews of Intervention. Three reviewers (SS, HF, MOP) screened the titles and abstracts using a single review process and the full text using a double review process. Any conflict was resolved and gained consensus with the involvement of a fourth reviewer (ALC).

All study types were included if they provided a statistical estimate of cancer mortality [e.g. standardized mortality ratio (SMR), hazard ratio (HR), relative risk (RR)], cancer incidence [e.g. standardised incidence ratio (SIR)], or cancer risk [e.g. HR, odds ratio (OR)] following pharmaceutical opioid exposure [defined by the World Health Organisation (WHO) Anatomical Therapeutic Chemical classification (ATC) code N02A] irrespective of their use and administration route. Studies were excluded if the study population had known cancer prior to opioid exposure, solely focused on the non-pharmaceutical use of opioids such as opium and heroin, or if no comparison was made regarding cancer incidence/cancer-related mortality and opioid exposure. We also excluded *in-vitro*/animal studies, reviews, grey literature, methodology papers, studies in languages other than English, conference abstracts, and studies whose full text were not available.

### Data extraction and synthesis

A purpose-built data extraction form was used by two reviewers (SS, HF) to independently extract data on study characteristics (publication details, location, study design, participant details, types of opioids, reason for opioid use/exposure, comparator, follow up/study period, cancer-related outcomes and key study findings from each included study. A third reviewer (MO) reviewed the data, and any conflicts were resolved by fourth reviewer (ALC). Because the studies included in this review showed considerable heterogeneity in terms of exposure, population and outcome measures, a descriptive narrative synthesis was undertaken rather than a meta-analysis.

### Quality appraisal

The Newcastle–Ottawa Scale (NOS) was used to assess the quality of the cohort and case–control studies. The NOS scores were calculated across three domains – selection, comparability and outcome (for cohort studies) or exposure (for case control studies). The NOS scores of 0–3, 4–6 and 7–9 were considered as low, moderate and high quality respectively.

## Results

### Study selection

**Fig. 1:**
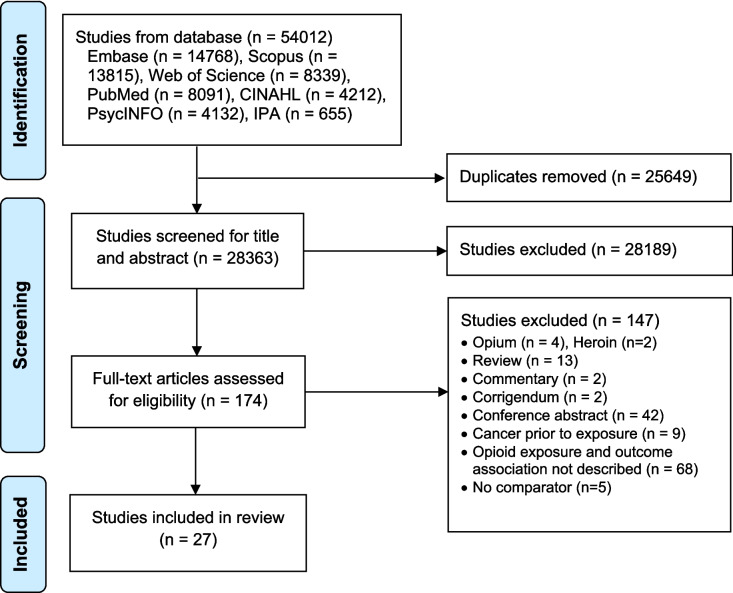
Prisma flow chart

A total of 54,012 studies were identified from the seven databases. After removing 25,649 duplicates, 28,363 studies were screened for title and abstract, identifying 174 of them as eligible for full-text screening. Subsequently, 27 studies met the inclusion criteria of the study (Fig. 1). A list of excluded studies with justification is provided in Appendix 2.

### Study characteristics

This review represented a total of 4,542,745 participants from two case–control studies (416,031 participants consisting of approximately 38% cancer cases in case control studies), four prospective cohort studies (509,284 participants) and 21 retrospective cohort studies (3,617,430 participants) (Table 1). The case–control studies [29, 30] were conducted in populations from United States and United Kingdom. The cohort studies represented populations from Australia (*n = *5) [31–35], US (*n = *5) [36–40], Norway (*n = *3) [41–43], South Korea (*n = *3) [44–46], Taiwan (*n = *3) [47–49], Israel (*n = *2) [50, 51], Denmark (*n = *2) [41, 52], UK (*n = *2) [53, 54], Italy (*n = *1) [55] and Czechia (*n = *1) [41]. One of the prospective cohort studies [41] used data from three countries – Czechia, Denmark and Norway. The study duration ranged from four years [53] to 29 years [41] with an average of 13 years.

**Table 1:** Studies according to design

Design	Author (year)
Prospective cohort (*n = *4)	Bjornaas (2008) [42];Eide (2023) [41]; Ekholm (2014) [52]; Macfarlane (2020) [53]
Retrospective cohort (*n = *21)	Bargagli (2001) [55]; Chang (2015) [47]; Degenhardt (2013) [31]; Gibson (2011) [32]; Hser (2019) [36]; Kostovski (2024) [43]; Maxwell (2005) [37]; Randall (2011) [33]; Rosca (2012) [51]; Larney (2015) [38]; Lee (2021) [48]; Song (2022) [44]; Zeng (2019) [54]; Olfson (2018) [39]; Veldhuizen (2014) [40]; Oh (2019) [45]; Oh (2020) [46]; Sun (2022) [49]; Grinshpoon (2011) [50]; Kelty (2017) [34]; Swart (2012) [35]
Case–control (*n = *2)	Havidich (2021) [29]; *Houston (2023) [30]

*nested case–control

### Quality assessment

NOS score ranked 12 of the 27 studies as high quality (Tables 2, 3, and 4 and Supplementary Table S1 and S2). This included 10 cohort studies [34, 44–46, 48–50, 52–54] (Table 2 and 3) and two case–control studies [29, 30] (Table 4). Of the remaining 15 cohort studies, 14 were of moderate quality [31–33, 35–41, 43, 47, 51, 55] and one of low quality [42]. Two cohort studies that were ranked high quality [48, 50] considered the general population as comparator (non-exposed cohort drawn from a different source). This was consistent with 12 cohort studies that were ranked moderate quality [31–33, 35, 37, 39–41, 43, 47, 51, 55]. Only one [43] of the moderate quality cohort studies demonstrated that the outcome of interest was not present at the start of the study and most of their cohorts were not compared on the basis of the design or analysis controlled for confounders [31–33, 35, 37, 40–43, 51, 55]. Studies that estimated standardized cancer-related mortality or incidence rates control for age and sex. These studies are unable to adjust for confounders such as exposure to cigarette smoking, alcohol or socioeconomic factors due to the methods used and the lack of access to individual-level data.

**Table 2:** Findings from cohort studies related to mortality (*n = *19)

Author (year), design	No. of participants (study duration)	Opioids	Use or Exposure	Comparator	Cancer type	All-cause mortality in OU, n	Cancer-related mortality			Study quality (NOS score)
							*n* (% of total OU or NOU)	SMR (95% CI)	HR or RR (95% CI)	
Ekholm (2014), Prospective [52]	13,127 (11 y) – [OU: 542—LTOU: 167, STOU: 375]	N02A (opioids), R05DA04 (codeine)	Chronic non-cancer pain	NOU1 (n: 2,015) and NOU2 (n: 10,570)	All	123 (LTOU: 47, STOU: 76)	OU: 32 (*5.9%) [LTOU: 12, STOU: 20] NOU: 335 (*2.7%) [NOU1: 85, NOU2: 250]	-	Age adjusted HR (95% CI) compared to NOU2 in LTOU: 1.63 (0.91–2.92), STOU: 1.28 (0.81–2.03), NOU1: 1.26 (0.98–1.61)	High (9/9)
Macfarlane (2020), Prospective [53]	466,486 (4 y) – [OU: 25,864]	**Weak opioids:** codeine, dihydrocodeine, meptazinol, pethidine, dextropropoxyphene, tramadol **Strong opioids:** morphine, buprenorphine, oxycodone, fentanyl, hydromorphine	Prescription	NOU	All	1,919 (Weak OU = 1,336, Strong OU = 583)	OU: *748 (*2.90%) NOU: *7,692 (*1.7%)	-	-	High (9/9)
Song (2022), Retrospective [44]	1,804,019 (9 y) – [LTOU: 8,320]	Not specified	Prescription for chronic pain	NOU	All	1,953	OU: 371 (*4.5%) NOU: 28,207 (*1.6%)	-	Multifactor adjusted HR (95% CI) compared to NOU in LTOU: 1.19 (1.02–1.40, *p* = 0.041) for 10-year mortality	High (8/9)
Zeng 2019, Retrospective [54]	88,902 (16 y) – [Tramadol: 44,451, Codeine: 16,922]	Tramadol, codeine	Prescription	Naproxen (n: 12,397), diclofenac (n: 6,512), celecoxib (n: 5,674), etoricoxib (n: 2,946), codeine (n:16,922)	All	1819 (tramadol: 1,267, codeine: 552)	OU: 394 (*0.6%) [tramadol: 287, codeine: 107] NOU: 81 (*0.3%) [naproxen: 35, diclofenac: 26, celecoxib: 13, etoricoxib: 7]	-	Multifactor adjusted HR (95% CI) in Tramadol compared to: Naproxen: 1.86 (1.24–2.81), diclofenac: 2.10 (1.33–3.34), celecoxib: 2.93 (1.57–5.47), etoricoxib: 2.16 (0.89–5.28), codeine: 1.04 (0.8–1.36)	High (8/9)
Lee (2021), Retrospective [48]	12,990 (12 y) – [LTOU: all]	Morphine, fentanyl, codeine, tramadol	Prescription	General population	All	558	OU: 13 (*0.1%)	0.29 (0.16–0.47)	-	High (7/9)
Oh (2019), Retrospective [45]	822,214 (13 y) – [weak OU: 48,286, strong OU: 1,143]	**Weak opioids:** codeine, dihydrocodeine, hydrocodone, tramadol **Strong opioids:** fentanyl, morphine, oxycodone, hydromorphone, methadone	Prescription for chronic pain	NOU (n: 772,785)	All	20,991	OU: 4,924 (*10.0%)	-	Multifactor adjusted HR (95% CI) compared to NOU in weak OU: 0.93 (0.89–0.96, *p* < 0.001), strong OU: 1.45 (1.28–1.64, *p* < 0.001)	High (7/9)
Chang (2015), Retrospective [47]	1,283 (6 y) – [OST: 983, non-OST with OUD: 300]	Heroin, methadone, buprenorphine	OST or Recreational (non-OST with OUD)	General population	All	102 (OST: 68, non-OST with OUD: 34)	OU: 15 (*1.2%) [OST: 10, non-OST with OUD: 5]	OST: 3.6 (1.7–6.6), non-OST with OUD: 5.5 (1.7–12.9)	-	Moderate (6/9)
Kostovksi (2024), Retrospective [43]	19,651 (8 y)	Methadone, Buprenorphine, Morphine	OST	General population	^#^All	2,383	OU: 192 (*1.0%) [digestive organs: 84, lung: 63, liver: 34, colon: 15, haematopoietic tissue: 11, pharynx: 7, oesophagus: 5, rectosigmoid: 6]	2.3 (2.0–2.7) [liver: 12.3 (8.5–17.2), lung: 3.9 (3.0–5.0), pancreas: 3.0 (1.7–5.0), oesophagus: 2.3 (0.7–5.4), colon: 1. 9 (1.1–3.1), rectosigmoid: 1.8 (0.7–4.0)]	-	Moderate (6/9)
Larney (2015), Retrospective [38]	96,878 (11 y) – [Old OUD ≥ 50 yrs: 36,608]	Not specified	Chronic non-cancer pain	Young OUD ≤ 50 yrs (n: 23,662), Old non-OUD (n: 36,608)	All	9,386 (Old OUD: 6,754, Young OUD: 2,632)	OU: 1,823 (*3.0%) [Old OUD: 1,518, Young OUD: 305] Old non-OUD: 1,240 (*3.4%)]	-	RR (95% CI) in older OUD compared to: Young OUD: 4.3 (4.1–4.5), Old non-OUD: 1.3 (1.2–1.3)	Moderate (6/9)
Olfson (2018), Retrospective [39]	76,325 (6 y)	Not specified	Recreational	General population	All	5,194	OU: 536 (*0.7%)	8.8 (8.0–9.5)	-	Moderate (6/9)
Bargagli (2001), Retrospective [55]	11,432 (17 y)	Methadone	OST	General population	All	1,734	OU: 39 (*0.3%)	2.4 (1.7–3.3)	-	Moderate (5/9)
Degenhardt (2013), Retrospective [31]	43,789 (20 y)	Methadone or Buprenorphine	OST	General population	All	3685	OU: 212 (0.5%)	1.7 (1.4–1.9)	-	Moderate (5/9)
Eide (2023), Prospective [41]	*29,486 [CZ: 5,217 (29 y), DK: 1,288 (18 y), NO: 11,389 (9 y)]	Methadone or Buprenorphine ± naloxone	OST	General population (< 65 yrs)	Neoplasm (C00-D49, ICD-10)	*5,322 (CZ: 421; DK: 3,389; NO: 1,512)	OU: *523 (*1.8%) [CZ: 17; DK: 331; NO: 175]	CZ (F: 0.3, M: 2.4), DK (F: 4.5, M: 6.9), NO (F: 25.5, M: 6.8)	-	Moderate (5/9)
Gibson (2011), Retrospective [32]	2,489 (26 y)	Methadone	OST	General population	All and Liver	478	OU: 60 (*2.4%) [liver: 13]	10.3 (7.9–13.3) [liver: 2.3 (1.2–3.9)]	-	Moderate (5/9)
Maxwell (2005), Retrospective [37]	13,264 (8 y)	Methadone	OST	General population	All	766	OU: 51 (*0.4%)	0.3 (0.3–0.3)	-	Moderate (5/9)
Randall (2011), Retrospective [33]	43,789 (20 y)	Not specified	OST	General population	All including lung, liver, skin, colorectal, non-Hodgkin’s lymphoma, leukemia, anogenital, pancreas, breast, kidney	3,533	OU: 212 (0.5%) [lung: 60, liver: 22, skin: 16, colorectal: 12, non-Hodgkin’s lymphoma: 12, leukemia: 10, anogenital: 9, pancreas: 9, breast: 5, kidney: 4]	1.7 (1.4–1.9) [lung: 3.6 (2.8–4.6), liver: 6.9 (4.3–10.5), skin:1.3 (0.7–2.1), colorectal: 0.9 (0.5–1.7), non-Hodgkin's lymphoma: 1.5 (0.8–2.6), leukemia: 1.3 (0.6–2.4), anogenital: 2.8 (1.3–5.3), pancreas: 2.1 (1.0–4.0), breast: 0.4 (0.1–0.9), kidney: 1.4 (0.4–3.7)]	-	Moderate (6/9)
Rosca (2012), Retrospective [51]	9,818 (9 y)	Methadone	OST	General population	All	960	OU: 96 (1.0%)	3.9 (3.1–4.7)	-	Moderate (5/9)
Veldhuizen (2014), Retrospective [40]	68,066 (15 y)	Not specified	Recreational	General population	All	13,107	OU: 2,473 (3.6%)	3.9 (3.8–4.1)	-	Moderate (5/9)
Bjornaas (2008), Prospective [42]	185 (20 y)	Not specified	Recreational	General population	All	70	OU: 3 (1.6%)	4.3 (1.4–13.5)	-	Low (3/9)

*Calculated, ^#^All including digestive organs, liver, colon, rectum, pancreas, oesophagus, stomach, respiratory organs, lung, epiglottis, lymphoid, kidney, skin, urinary tract, pharynx, tonsil, CNS, thyroid

Abbreviations: *CI* Confidence interval, *CNS* Central nervous system, *CZ* Czechia, *F* Female, *HR* Hazard ratio, *DK* Denmark, *LTOU* Long term opioid users, *M* Male, *NO *Norway, *NOU* Non-opioid users, *NOU1* Non-opioid users with chronic pain, *NOU2* Non-opioid users without pain, *OST* Opioid substitution therapy, OU Opioid users, *OUD *Opioid use disorder, *RR* Risk ratio, *SMR* Standardised mortality ratio, *STOU* Short term opioid users, ; *y *years

Cancer related mortality data for general population was not available

**Table 3:** Findings from cohort studies related to cancer incidence (*n = *9)

Author (year), Design	No. of participants (study duration)	Opioids	Use or Exposure	Comparator	Cancer type	Cancer incidence		Study quality (NOS score)
						*n* (% of total OU or NOU)	SIR (95% CI) or HR (95% CI)	
Grinshpoon (2011), Retrospective [50]	^#^18,659 (17 y)	Methadone	OST	General population	Lung, Bladder & kidney, Lymphoma, Larynx, Liver, Leukaemia, Colorectal, Brain, Testis, Pancreas, Prostate, Breast, Cervix, Ovary	OU: 184 (*1.0%) [lung: 30, bladder/kidney: 19, lymphoma: 18, larynx: 19, liver: 10, leukaemia: 7, colorectal: 9, brain: 7, testis: 4, pancreas: 4, prostate: 2, breast: 4, cervix: 11, ovary: 2]	**SIR (95% CI)** M: 0.88 (0.76–1.0), F: 1.06 (0.76–1.36)	High (7/9)
Kostovski (2024), Retrospective [43]	19,651 (8 y)	Methadone, Buprenorphine, Morphine	OST	General population	All including digestive organs, liver, colon, rectum, pancreas, oesophagus, stomach, respiratory organs, lung, epiglottis, lymphoid, kidney, skin, urinary tract, pharynx, tonsil, CNS, thyroid	OU: 455 (*2.3%) **[**digestive organs: 137, liver: 43, colon: 28, rectum: 21, pancreas: 19, oesophagus: 6, stomach: 5, respiratory organs: 94, lung: 88, epiglottis: 6, lymphoid: 31, kidney: 18, skin (melanoma): 18, urinary tract: 16, pharynx: 16, tonsil: 11, CNS: 16, thyroid: 6, skin (non-melanoma): 5, prostate: 13, breast (female): 29, genitals (female): 26, cervix uteri: 11]	**SIR (95% CI)** **Overall: 1.19 (1.08–1.30**), Liver: 12.59 (9.11–16.95), epiglottis: 4.66 (1.70–10.15), trachea: 3.51 (2.81–4.32), pancreas: 2.59 (1.56–4.05), oesophagus: 1.85 (0.68–4.02), pharynx: 1.73 (0.99–2.82), kidney: 1.35 (0.80–2.14), rectum: 1.33 (0.82–2.04), colon: 1.13 (0.75–1.63), urinary tract: 1.12 (0.64–1.82), stomach: 1.04 (0.33–2.42), lymphoid: 0.87 (0.59–1.23), CNS: 0.82 (0.47–1.33), thyroid: 0.74 (0.27–1.61), melanoma: 0.54 (0.32–0.85), skin (non-melanoma): 0.49 (0.16–1.14), breast (female): 0.62 (0.41–0.89), prostate: 0.26 (0.14–0.44), genitals (female): 1.23 (0.80–1.80)	Moderate (6/9)
Swart (2012), Retrospective [35]	45,412 (22 y)	Not specified	OST	General population	Kaposi sarcoma, Liver, Lung, Larynx, Vulva, Anus SIR, Tonsil, Pancreas, Nasal Cavity, Cervix, Mouth, Oesophagus, Bladder, Tongue, Non-Hodgkin Lymphoma, Salivary gland, Lip, Stomach, Myeloid neoplasm, Small intestine, Hodgkin lymphoma, Ovary, Kidney, Testis, Non-melanoma skin, Nasopharynx, Melanoma (skin), Colorectal, Brain & CNS, Breast, Thyroid, Connective & soft tissue, Prostate	OU: 819 (1.8%) [803 first cancer and 16 second cancer]	**SIR (95% CI)** **Overall: 1.15 (1.07—1.23),** Kaposi sarcoma: 837 (383–1,589)], liver [8.04 (6.18–10.3)], lung [4.02 (3.32–4.82)], larynx [3.60 (1.86–6.28)], vulva [3.59 (1.17–8.38)], anus and anal canal [3.39 (1.63–6.24)], tonsil [3.38 (1.62–6.22)], pancreas [2.81 (1.72–4.34)], nasal cavity [2.38 (0.65–6.10)], cervix uteri [2.27 (1.61–3.08)], mouth [2.26 (0.98–4.46)], oesophagus [2.03 (0.93–3.85)], mesothelioma [1.85 (0.38–5.40)], bladder [1.81 (0.91–3.24)], tongue [1.68 (0.77–3.20)], non-Hodgkin lymphoma [1.51 (1.20–1.88)], salivary gland [1.45 (0.40–3.72)], lip [1.45 (0.88–2.23)], stomach [1.41 (0.81–2.26)], all myeloid neoplasms [1.38 (0.92–1.97)], small intestine [1.19 (0.25–3.48)], Hodgkin lymphoma [1.16 (0.67–1.85)], all malignant neoplasms [1.15 (1.07–1.23)], ovary [1.14 (0.52–2.16)], kidney [1.11 (0.69–1.67)], testis [1.08 (0.78–1.45)], non-melanoma skin [0.84 (0.27–1.96)], nasopharynx [0.76 (0.16–2.23)], melanoma (skin) [0.72 (0.59–0.87)], colorectal (excluding anus) [0.55 (0.37–0.78)], brain and CNS [0.50 (0.25–0.88)], breast [0.48 (0.35–0.62)], thyroid [0.41 (0.23–0.66)], connective and soft tissue [0.35 (0.07–1.04)], prostate [0.16 (0.06–0.32)]	Moderate (6/9)
Ekholm (2014), Prospective [52]	13,127 (11 y) – [LTOU: 167, STOU: 375]	N02A (opioids), R05DA04 (codeine)	Chronic non-cancer pain	NOU1 (n: 2,015) and NOU2 (n: 10,570)	All	OU: 49 (*9.0%)—[LTOU: 14, STOU: 35] NOU: 707 (*5.6%)—[NOU1: 155, NOU2: 552]	**HR (95% CI)** **Multivariate adjusted:** LTOU: 1.03 (0.61–1.77), STOU: 1.27 (0.90–1.79), NOU1: 1.21 (0.93–1.35), NOU2: 1	High (9/9)
Oh (2020), Retrospective [46]	351,701 (5 y) – [OU: 25,153 (Weak OU: 24,415, Strong OU: 712)]	Codeine, dihydrocodeine, hydrocodone, tramadol, fentanyl, morphine, oxycodone, hydromorphone, methadone	Prescription for chronic pain	NOU (n: 326,548)	Gastric, Oesophageal, Colorectal, Gall bladder & biliary tract, Head & neck, Brain, Liver, Pancreatic, Lung, Bone & articular cartilage, Neoplasm of breast and genitals, Urinary tract, Thyroid, Lymphoma or leukemia	OU: *1871 (*7.4%) **[**gastric: 1,748, oesophageal: 107, colorectal: 2,621, gall bladder & biliary tract: 451, head & neck: 128, brain: 102, liver: 3,525, pancreatic: 1,189, lung: 2,047, bone & articular cartilage: 53, neoplasm of breast and genitals: 5,696, urinary tract: 974, thyroid: 1,297, lymphoma or leukemia: 591] NOU: *20,217 (*6.2%)	**HR (95% CI)** **Overall: 1.20 (1.15–1.25),** Weak OU: 1.18 (1.13–1.23), Strong OU: 1.32 (1.10–1.59), gastric: 0.89 (0.76–1.05), oesophageal: 0.79 (0.40–1.54), colorectal: 1.02 (0.90–1.15), gall bladder & biliary tract: 1.01 (0.75–1.37), head & neck: 0.70 (0.38–1.30), brain: 0.81 (0.39–1.71), liver: 1.35 (1.22–1.50), pancreatic: 1.04 (0.86–1.24), lung: 1.19 (1.04–1.36), bone & articular cartilage: 0.92 (0.35–2.38), neoplasm of breast and genitals: 1.36 (1.26–1.47), urinary tract: 1.18 (0.98–1.43), thyroid: 1.48 (1.24–1.78), lymphoma or leukemia: 1.23 (0.96–1.58)	High (8/9)
Sun (2022), Retrospective [49]	63,610 (11 y) – [LTOU: 50,888]	Not specified	Prescription for chronic pain	NOU (n: 12,722)	Lung, Hepatocellular, Colorectal, Breast, Prostate, Head & Neck, Pancreatic, Gastric, Oesophageal, Ovarian, Others	**LTOU vs NOU** **4,191 (8.2%) vs 522 (4.1%),** [lung: 506 (1.0%) vs 67 (0.5%); hepatocellular: 599 (1.2%) vs 76 (0.6%); colorectal: 684 (1.3%) vs 71 (0.6%); breast: 380 (0.8%) vs 39 (0.3%); prostate: 217 (0.4%) vs 27 (0.2%); head & Neck: 150 (0.3%) vs 21 (0.2%); pancreatic: 79 (0.2%) vs 38 (0.3%); gastric: 184 (0.4%) vs 19 (0.2%); oesophageal: 66 (0.1%) vs 9 (0.1%); ovarian: 56 (0.1%) vs 6 (0.1%); others: 1700 (3.3%) vs 211 (1.7%)]	**HR (95% CI) – Multivariate adjusted:** **Overall: 2.66 (1.44–2.94),** Lung: 2.63 (2–3.45); hepatocellular: 2.63 (2.04–3.33); colorectal: 3.13 (2.5–4.0); breast: 3.23 (2.27–4.35); prostate: 2.85 (1.92–4.0); head & Neck: 2.22 (1.41–3.45); pancreatic: 1.52 (1.09–2.50); gastric: 3.23 (2.04–5.26); oesophageal: 2.5 (1.23–5.0); ovarian: 3.03 (1.32–7.14); thers: 2.63 (2.27–3.03)	High (8/9)
Kelty et al. (2017), Retrospective [34]	15,411 (9 y)	Methadone (n: 2,227), Buprenorphine (n: 1,952), Naltrexone (n: 958)	OST	Any opioid (n: 5,137), Control (n: 5,137)	Lip, oral cavity & pharynx; Digestive Respiratory; Bone & cartilage; Melanoma & skin; Mesothelial and soft tissue; Breast; Female genital; Male genital; Urinary tract; Eye, brain & CNS; Thyroid; Lymphoid	**Any opioid vs control** **45 (0.9%) vs 67 (1.3%)** **[**Lip, oral cavity & pharynx: 10 vs 17; digestive organs: 4 vs 4; respiratory organs: 7 vs 2; melanoma and skin: 9 vs 18; mesothelial and soft tissue: 1 vs 3; breast (female): 4 vs 6; genital organs (female): 4 vs 3; genital organs (male): 1 vs 3; eye, brain & CNS: 1 vs 2; lymphoid: 4 vs 8] **Methadone vs buprenorphine vs naltrexone** 20 (0.9%) vs 13 (0.7%) vs 6 (0.6%) **Methadone vs naltrexone** Respiratory organs: 5 (0.2%) vs 2 (0.2%)	**HR (95% CI)** **Overall: 0.69 (0.47–1.00);** Methadone: 0.81 (0.49–1.34) [with respiratory: 7.53 (1.46–38.93)]; buprenorphine: 0.74 (0.41–1.33); naltrexone: 0.65 (0.28–1.50) [with respiratory: 7.65 (1.07–54.48)]	High (7/9)
Oh (2019), Retrospective [45]	822,214 (13 y) – [weak OU: 48,286, strong OU: 1,143]	Codeine, dihydrocodeine, hydrocodone, tramadol, fentanyl, morphine, oxycodone, hydromorphone, methadone	Prescription for chronic pain	NOU (n: 772,785)	All	**OU: 11,737 (23.7%)** [Weak OU: 11,516 (23.8%), Strong OU: 221 (19.3%)] **NOU: 39,597 (5.1%)**	-	High (7/9)
Hser (2019), Retrospective [36]	7,728 (8 y) – [OUD: 2,576]	All opioids except buprenorphine	OST	non-OUD (n: 5,152)	All	OUD: *363 (14.1%) **non-OUD**: *670 (13.0%)	-	Moderate (6/9)

^#^87.4% male *Calculated

Abbreviations: *CI* confidence interval, *CNS* Central nervous system, *HR* Hazard ratio, *LTOU *Long term opioid users, *NOU *Non-opioid users, *NOU1* Non-opioid users with chronic pain, *NOU2* Non-opioid users without pain, *OST* Opioid substitution therapy, *OU* Opioid users, *OUD* Opioid use disorder, *SIR* Standardised incidence ratio, *STOU* Short term opioid users, *y* years

N02A and R05DA04 are Anatomical Therapeutic Chemical classification codes

**Table 4:** Findings from case–control studies (*n = *2)

Author (year)	No. of participants (study duration)	Opioids	Case (Cancer group), *n* (%)	Control (Non-cancer group), *n* (%)	Exposed group (OU), *n* (%)	Non-exposed group (NOU), *n* (%)	Exposed cases (OU with cancer), *n* (%)	Non-exposed cases (OU without cancer), *n* (%)	Cancer risk, OR (95% CI)	Study quality
Havidich (2021) [29]	348,319 (5 y)	Not specified	**143,921 (41.3%)** (Bladder = 11,623, Brain = 2,678, Breast = 34,123, Colon = 24,540, Oesophagus = 2,311, Kidney = 6,718, Leukemia = 6,725, Liver = 4,457, Lung = 40,311, Lymphoma = 10,435	204,398 (58.7%)	*118,459 (34.0%)	*229,860 (66.0%)	50,647 (35.2%)	67,812 (33.2%)	Overall = 1.01 (0.99–1.03), Breast = 0.96 (0.92–0.99), Colon = 0.90 (0.86–0.93), Lung = 1.04 (1.01–1.07), Liver = 1.19 (1.08–1.21)	high
^#^Houston (2023) [30]	*67,712 (12 y)	Codeine, dihydrocodeine	*12,625 Oesophageal = 2,432, Gastric = 1,443, Colorectal = 8,750	*55,087 Oesophageal = 10,590, Gastric = 6,233, Colorectal = 38,264	*21,855 (32.3%) (oesophageal = *4,242, Gastric = *2,723, Colorectal = *14,890)	*45,857 (67.7%) (Oesophageal = *8,780, Gastric = *4,953, Colorectal = *32,124)	*4,219 (Oesophageal = *869, Gastric = *577, Colorectal = *2,773)	*17,636 (Oesophageal = 3,373, Gastric = 2,146, Colorectal = 12,117)	**Codeine only** Oesophageal = 1.12 (1.00–1.25), Gastric = 1.29 (1.12–1.50), Colorectal = 0.96 (0.90–1.02) **Dihydrocodeine only** Oesophageal = 1.06 (0.92–1.23), Gastric = 1.10 (0.92–1.32), Colorectal = 0.94 (0.87–1.02) **Overall (either/or)** Oesophageal = 1.16 (1.04–1.29), Gastric = 1.26 (1.10 −1.45), Colorectal = 0.96 (0.90–1.02)	high

^#^Nested case control; *Calculated

Abbreviations: *CI* confidence interval, *LTOU *Long term opioid users, *NOU *Non-opioid users, *NOU1 *Non-opioid users with chronic pain, *NOU2 *Non-opioid users without pain, *OR* Odds ratio, *OST* Opioid substitution therapy, *OU* Opioid isers, *OUD *Opioid use disorder, *STOU* Short term opioid users, *y *years

Cohort studies with in-study comparator groups were able to adjust for a wider range of variables (which included known or suspected confounders). Confounders were addressed either by matching variables across the comparator(s) or statistically using propensity score matching and regression modelling. Overall, most studies (*n = *23) matched/adjusted age and sex, followed by comorbidities-related factors (*n = *9), residence (*n = *6), race/ethnicity (*n = *5), income (*n = *5), smoking status (*n = *5) and alcohol use (*n = *4). Two studies considered other variables such as education, body mass index, calendar year, year of registration, employment status, deprivation and non-opioid medication use. There was one study for cohabitation status, year of immigration, GP practice, history of cancer, pain status, hospital visits, surgery, disability, viral status (HCV, HBV, HIV) and health care utilisation.

### Types of opioids

Ten studies did not specify which opioid was used [34], one of which mentioned all opioids were considered except buprenorphine [41]. The remaining 17 studies either used weak opioids (codeine, dihydrocodeine, meptazinol, dextropropoxyphene, hydrocodone, tramadol), strong opioids (morphine, pethidine, buprenorphine, oxycodone, fentanyl, hydromorphine, hydromorphone and methadone) or opioid antagonists (naloxone and naltrexone). The most commonly used strong opioid was methadone [31, 32, 34, 37, 41, 43, 45–47, 50, 51, 55] followed by buprenorphine [31, 34, 41, 43, 47, 53], tramadol [45, 46, 48, 53, 54], fentanyl [45, 46, 48, 53] and morphine [43, 45, 46, 48, 53]. Codeine [30, 45, 46, 52, 53] and dihydrocodeine [30, 45, 46, 53] were the most common weak opioids. The use of an opioid antagonist was less common – one study explicitly used naltrexone [34] while the other used naloxone in combination with buprenorphine [41].

### Opioid exposure and comparator

In terms of cohort studies, the main reason for exposure to opioids was opioid substitution therapy (*n = *13) [31–37, 41, 43, 47, 50, 51, 55], followed by chronic pain (*n = *9) [38, 44–46, 48, 49, 52–54] and recreational use (*n = *3) [39, 40, 42]. The comparator was the general population (14 studies) [31–33, 35–37, 39–43, 48, 50, 51] or non-opioid users (11 studies) [34, 36, 38, 44–47, 49, 52–54] sometimes specified as having or not having chronic pain, or users of other analgesics (two studies) [34, 54].

### Overall outcome measures

This review focused on three cancer-related outcome measures – for cohort studies the outcomes of interest were “cancer mortality” and “cancer incidence”, while for case–control studies the outcome of interest was “cancer risk”. Out of 19 cohort studies reporting cancer-related mortality, the majority (*n = *14, 73.7%) reported an increased SMR or HR with opioid use. There were two studies (10.5%) that reported no change between groups, three (15.8%) reported a decrease and one (5.3%) reported a contrasting effect between week and strong opioids (participants receiving weak opioids had a HR less than one, and participants receiving strong opioids had a HR greater than 1) (Table 2). Within the nine cohort studies related to cancer incidence, five (55.6%) studies reported an overall increase in cancer incidence with opioid use, two further studies (22.2%) revealed that even though aggregated data did not show such an increase, there were organ-specific and/or opioid-specific increases (or decreases) in cancer incidence in participants exposed to opioids. The only prospective study reporting cancer incidence found no change in cancer incidence in participants exposed to opioids (Table 3). Findings from the two case–control studies varied according to body site-specific cancer risk (Table 4).

#### Cancer-related mortality

We identified four types of statistical estimates reporting on cancer-related mortality from 19 cohort studies (Table 2) – prevalence percentage, standardised mortality ratio, hazard ratio and relative risk.

**Fig. 2:**
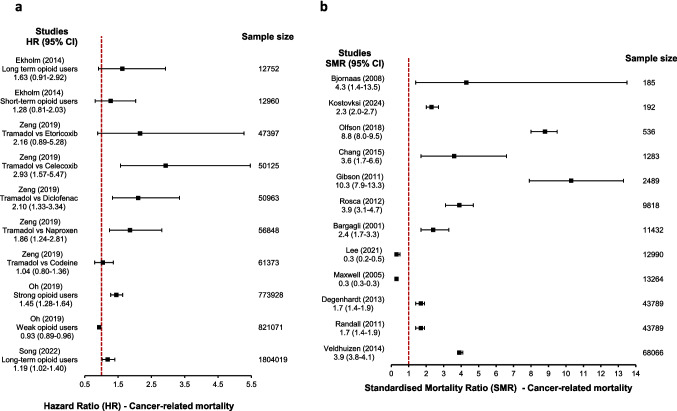
**a**, Summary plot of observed effect sizes showing hazard ratio of cancer-related mortality according to types of opioid users. The sample size indicated is the total of participants in the two groups compared; **b**, Summary plot of observed effect sizes showing SMR of cancer-related mortality. Sample size refers to the number of participants contributing to the SMR calculation

- *Prevalence percentage:* There were 19 studies that reported on the cancer mortality (Table 2). The overall median (q1-q3) prevalence of cancer mortality (in those using opioids) derived from all the studies was 1.2% (0.6–3.0%) (supplementary table S3).
- *Hazard ratio:* Four studies reported on hazard ratio (HR) of cancer-related mortality [44, 45, 52, 54] in patients exposed to opioids due to chronic pain. Three of these studies [44, 45, 52] compared different types of opioid users with non-opioid users as a comparator and their findings are presented in Table 2 and Fig. 2a. Oh *et al.* [52] compared participants exposed to strong and weak opioids to non-opioid users with and without pain; non-opioid users without pain were the reference comparator. Strong opioid users had an increased HR for cancer-related mortality and weak opioid users had a reduced HR for cancer-related mortality. Song *et al.* [44] and Elkholm *et al.* [52] compared long-term opioid users and long and short-term opioid uses to non-opioid users respectively. These studies report a HR point estimate greater than one but not reach statistical significance. Zeng *et al. *[54]*.* compared tramadol with five different groups – four non-steroidal antiinflammatory drugs (naproxen, diclofenac, celecoxib and etoricoxib) and codeine. This propensity-matched study suggested that tramadol users had higher risk of cancer-related mortality compared to all non-steroidal antiinflammatory drugs. This was statistically significant when compared to naproxen (HR 1.86, 95% CI 1.24–2.81), diclofenac (HR 2.10, 95% CI 1.33–3.34), celecoxib (HR 2.93, 95% CI 1.57–5.47) but not with etoricoxib (HR 2.16, 95% CI 0.89–5.28).
- *Relative risk:* One study that reported relative risk (RR) [38] found that opioid users who were 50 years and above (old opioid users) had 4.3 times high risk of cancer-related mortality at statistically significant level (95% CI 4.1–4.5) compared with those below 50 years of age (young opioid users) aged below (Table 2). This population also had statistically significant high risk of cancer-related mortality (RR 1.3, 95% CI 1.2–1.3) compared to the non-opioid users of similar age (older non-opioid users).
- *Standardised mortality ratio:* There were 13 studies that reported on the SMR related to cancer (Table 2). One of the 13 studies reported separate SMR for male and female but not an overall SMR [41]. This study reported that males from three European countries who were on OST had higher cancer-related mortality than the general population (Czechia: 2.4, Denmark: 6.9 and Norway: 6.8) but this was not consistent among females (Norway: 25.5, Denmark: 4.5, Czechia: 0.3). Of the remaining studies (Fig. 2b), 10 showed a higher standardized cancer-related mortality compared to the general population (SMR ranging from 1.7 [31, 33] to 10.3 [32]), and two studies reported a lower standardized cancer-related mortality (SMR 0.3) [37, 48].

#### Cancer incidence

Our review identified three types of statistical estimates reporting on cancer incidence from nine cohort studies (Table 3) – incidence percentage, standardised incidence ratio and hazard ratio.

**Fig. 3:**
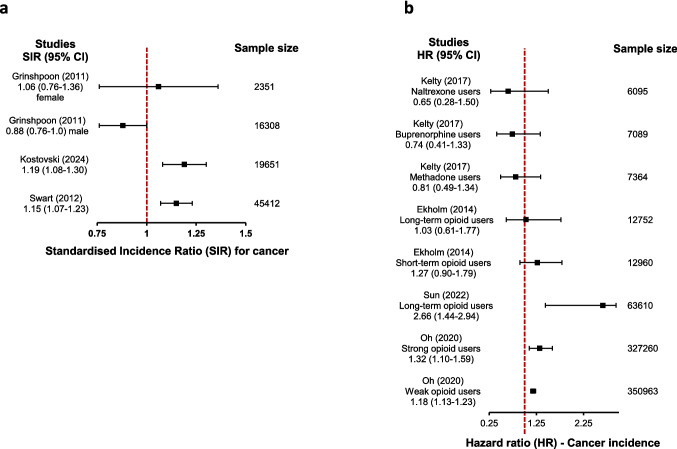
**a**, Summary plot of observed effect sizes showing standardised cancer incidence ratio. Sample size refers to the number of participants contributing to the SIR calculation; **b**, Summary plot of observed effect sizes showing estimate of cancer hazard in different opioid users. The sample size indicated is the total of participants in the two groups compared

- *Incidence percentage:* Nine studies reported on the cancer incidence. The overall median (q1-q3) incidence of cancer was found to be 7.4% (1.8–9.0%) (supplementary table S4). This was very low in studies with general population as comparator (1.8%) compared to studies with non-opioid users as comparator (8.6%).
- *Standardised incidence ratio:* Three studies reported on SIR for cancer with one study [50] providing data according to sex only (Table 3, Fig. 3a). This study reported point estimates in different directions (SIR > 1 for female participants and < 1 for male participants), but neither values were statistically significant. The other two studies [35, 43] showed that opioid users had 15–19% higher incidence of cancer compared to the general population, which was statistically significant.
- *Hazard ratio:* Four studies [34, 46, 49, 52] reported on hazard ratio for cancer incidence (Fig. 3b). One study reported hazard ratios for cancer incidence < 1 for users of methadone, buprenorphine and naltrexone compared to non-opioid uses, but none of these reached statistical significance [34]. Another study that adjusted for multiple variables in a regression model observed that long-term opioid users were 2.66 times more likely to develop cancer compared to the non-opioid users (95% CI 1.44–2.94) [49]. Elkholm *et al.* [52] conducted a multivariate regression analysis suggested that this effect was not significant (HR 1.03, 95% CI 0.61–1.77) [52]. Oh *et al*. [46] reported that the hazard of experiencing cancer incidence was significantly higher but modest in both the weak opioid users (HR 1.18, 95% CI 1.13–1.23) and strong opioid users (HR 1.32, 95% CI 1.10–1.59) compared to non-opioid users [46].

#### Cancer risk

Two case–control studies reported the odds of cancer (Table 4). In one study, while the odds of overall cancer was null, the risks of lung cancer (OR 1.04, 95% CI 1.01–1.07) and liver cancer (OR 1.19 95% CI 1.08–1.21) were higher, and the risks of breast cancer (OR 0.96, 95% CI 0.92–0.99) and colon cancer (OR 0.90, 95% CI 0.0.86–0.93) were lower among opioid users than non-opioid users [29]. In the second study, the odds of oesophageal cancer (OR 1.16 95% 1.04–1.29) and gastric cancer (OR 1.26 95% 1.10–1.45) were significantly higher among users vs. non-users of codeine and/or dihydrocodeine [30].

### Organ site-specificity

Whether they report an increased or unchanged aggregated risk of cancer incidence or mortality, several studies reported organ-specific differences (Table 2, 3, 4). Lung [29, 33–35, 43, 46, 49, 50] and liver cancer risk [29, 32, 33, 35, 43, 46, 49, 50] were increased in all studies that evaluated them. Pancreatic cancer risk was increased in most [33, 35, 43, 49] but not all (no change in[46]) of studies that tested it. Gastric and oesophageal cancer risks were increased in two studies [30, 49] but not significantly in three others [35, 43, 46]. Depending on studies, colorectal cancer risk was decreased [29, 35, 50] unaltered [30, 33, 43, 46] or increased [49]. Interestingly, breast cancer risk was decreased in most studies [29, 33, 35, 43, 50] (increased in [49]).

### Opioid drug specificity

Opioid maintenance or substitution therapy involves the administration of opioid agonists [such as the long-acting mu opioid receptor (MOR) agonist methadone, or the partial MOR agonist buprenorphine] or antagonists (such as the predominantly MOR antagonist naltrexone). In their retrospective cohort study comparing cancer incidence between people treated with methadone, buprenorphine or naltrexone for opioid dependence, Kelty *et al.* showed similar trends between the three treatment options and the sex and age-matched control cohort drawn from the electoral roll [HR (95% CI) 0.81 (0.49–1.34; 0.74 (0.41–1.33); 0.65 (0.28–1.50] for aggregated body sites. In contrast, increased respiratory cancer incidence was reported in methadone and naltrexone users, while there was no increase in respiratory cancer incidence in buprenorphine-treated patients [34]. In another study comparing the effects of different drugs, the cancer mortality of participants taking tramadol was higher than that of participants taking anti-inflammatory drugs such as naproxen [HR (95% CI 1.86 (1.24–2.81)], diclofenac [HR (95% CI) 2.10 (1.33–3.34)], or celecoxib [HR (95% CI) 2.93 (1.57–5.47)], no difference was observed in comparison with codeine [54].

### Intensity of exposure

Proxy measures for the intensity of opioid exposure include the duration of exposure, or the strength of the opioids taken by participants. Ekholm *et al.* compared the cancer incidence in long term (used at least one prescription/month for six months over the past year) or short term (used at least one prescription over the past year and were not long term) users to non-opioid users with no pain, and found that neither long term nor short term exposure increased the HR [1.03 (0.6–1.77) and 1.27 (0.90–1.79)] [52]. In term of opioid strength, in their retrospective cohort study assessing cancer mortality [45], Oh et al. observed that users of weak opioids (codeine, dihydrocodeine, hydrocodone or tramadol) had a decreased HR compared to non-opioid users [0.93 (0.89–0.96)] whereas users of strong opioids (fentanyl, morphine, oxycodone, hydromorphone, methadone) had an increased HR compared to non-opioid users [1.45 (1.28–1.64)]. In contrast, in their retrospective cohort study assessing incidence[46], weak and strong opioids alike increased HR compared to non-opioid users [1.18 (1.13–1.23 and 1.32 (1.10–1.59)], posing the question of a different effect of opioid strength on incidence and mortality.

### Role of pain

In their prospective cohort study, Ekholm *et al.* compared long-term opioid users, short-term opioid users and non-opioid users with pain to non-opioid users without pain [52]. Interestingly, non-opioid users with pain had a HR similar to that of the short-term opioid users for cancer incidence [1.21 (0.93–1.35) *vs* 1.27 (0.90–1.79)] as well as cancer mortality [1.326 (0.98–1.61) *vs* 1.28 (0.81–2.03)]. Houston et al. compared the weak opioids codeine and dihydrocodeine to other analgesics in influencing the risk of gastrointestinal cancers [30]. While weak opioids increased the risk of gastric cancer compared to non-opioid users [adjusted OR 1.26 (1.10–1.45) *p* = 0.001], this association was attenuated when weak opioid use were compared with paracetamol use [adjusted OR 1.09 (0.86, 1.39) *p* = 0.45]. Similarly, weak opioid users had an increased risk of oesophageal cancer compared to non-opioid users [adjusted OR 1.16, (1.04–1.29) *p* = 0.01), but this association was attenuated when weak opioid users were compared with ibuprofen users [adjusted OR 1.03 (0.86, 1.22) *p* = 0.76].

## Discussion

There are important clinical questions regarding whether the use of opioids influence the risks of cancer incidence and cancer mortality. This study sought to address these questions by synthesising data from studies that measured cancer incidence and/or mortality in participants, without a prior diagnosis of cancer, who were exposed to opioid medications or recreational opioids. The majority of studies involved people receiving opioids for treatment of opioid use disorder; however, a number of studies focused on participants with chronic pain. Many studies estimated standardized cancer mortality or cancer incidence by comparing these outcomes among their participants with expected rates in the general population. These studies were designed to estimate rates of cancer mortality and/or cancer incidence in people with opioid use disorder, rather than directly measure the impact of opioid exposure on cancer outcomes. Consequently, these studies are unable to control for important confounding factors that might influence the relationship between opioid exposure and cancer outcomes, such as tobacco smoking, socioeconomic status, body mass index, and alcohol consumption. Studies that employed methods to address the confounding through study design or analysis (e.g. in-study comparator groups and multivariable analysis) were more likely to include participants with chronic pain rather than opioid use disorder. These studies tend to report a small increase in risk of cancer-related outcome, frequently with confidence intervals that include a null effect.

With these points in mind, we could summarise the key findings as follows: The available evidence is consistent with a range of hypotheses regarding the effect of opioid use on the risk of cancer incidence and cancer mortality. While the raw data reported in most studies suggest an association between exposure to opioids and a mild-moderate increase in risk of cancer incidence and/or mortality, there is uncertainty in these estimates due to risk of bias. The risk of bias is especially high in retrospective studies comparing cancer mortality or incidence in OST/OMT (opioid maintenance treatment) opioid users against the general population. The synthesised evidence does not indicate the existence of a large effect of opioids on cancer mortality and/or incidence. While some data reports that the risk of certain opium-related cancers, such as lung and pancreatic cancers, is elevated among opioid users, and that the risk of non opium-related cancers such as breast or colon cancer is lower in opioid users, the available evidence does not allow for a firm conclusion regarding organ-specific effects of opioids. Finally, the available evidence is limited in its ability to assess the effects of individual opioids on risk of cancer incidence and cancer mortality.

This is the first systematic review to synthesise the diverse epidemiological evidence available on the question of whether opioid exposure among cancer-free individuals is independently associated with the risk of future cancer incidence or cancer mortality. The findings highlight the substantial challenges in reliably estimating the effects of opioid exposure on cancer incidence and mortality. The factors that influence risk of bias in the available evidence are reviewed below.

### Confounders for increased cancer risk

#### OST/OMT

Patients have been reported to have nearly twice the risk to die of cancer compared to the general population [33], however it is interesting to note that patients in treatment have been reported to have a decreased cancer risk compared to patients out of treatment [56]. This is also apparent in the study by Chang et al. where all participants were heroin users, and the cancer SMR among non-OST patients was higher than that of the OST patients [47]. This provides evidence against there being a simple and direct effect of opioid agonism on tumour biology, and is further supported by the fact that opioid maintenance involves the administration of opioid agonists (e.g. methadone, buprenorphine) or antagonists (naltrexone). These observations point to characteristics or behaviours associated with this group of patients that increase the risk of cancer incidence or mortality. Indeed, alternative factors have been proposed to explain higher cancer risk in OST patients compared to the general population, including misclassification of symptoms, delayed diagnosis or delayed treatment in this disadvantaged group [33, 51].

#### Recreational drug use

Has been associated with cancer risk *via* other associated substances of abuse like tobacco and alcohol, and the likelihood to engage in high-risk behaviours such as unprotected sexual activities, sharing needles, which can increase exposure to viruses that could have carcinogenic effects. These confounders are developed below:

- Up to 95% of opioid users are tobacco smokers [57, 58] and smoking increases the risk of opioid user disorder, with a pooled odds ratio of opioid use disorders of 8.23 (3.07–22.09) for smokers compared to nonsmokers [59]. This is proposed to be explained by a bidirectional relationship between addiction and cigarette smoking [59]. Smoking is a major risk factor for lung cancer [60]. Except for two studies [29, 49], none of the studies in our review that showed increased lung cancer risk in relation to opioid use, had adjusted for smoking [33–35, 46, 50]. Some studies even discussed tobacco use as the likely explanation for the observed associations [33–35, 50]. Tobacco smoking is also associated with increased risk of several other cancers (urinary tract, upper aerodigestive tract, larynx, pancreas, nasopharynx, stomach, liver, kidney, uterine cervix and myeloid leukemia) [61].
- Similarly, most studies observing an association between the incidence or death from liver cancer and opioid exposure [32, 33, 35, 46, 50] attributed this increased risk to the prevalence of HCV/HBV amongst opioid users and/or alcohol consumption as a plausible mechanism. There is a high prevalence of excessive alcohol consumption in opioid users [62, 63] and alcohol abuse increases the risk of cancer in the liver and other sites [64, 65]. In addition, intravenous injection is a common route of administration among recreational opioid users, which includes practices that increase the risk of blood-borne viral infection and therefore is a significant predictor of testing hepatitis B (HBV) or C (HCV) positive [66–68]. HBV and HCV have been reported to have oncogenic potential and to be associated with an increase in the risk of hepatocellular carcinoma [69, 70].
- Two studies reveal an increased risk of anogenital cancer mortality [33] or incidence [35] in opioid users. Authors suggested an increased prevalence of HIV and human papilloma virus (HPV) infection in this cohort, resulting from a higher propensity to engage in risky practices/behaviours [71–73]. Oncogenic strains of HPV cause cancers of the cervix, vulva, vagina, penis, anus, the oral cavity and oropharynx [74], while HIV facilitates HPV infection, HPV persistence, and the development of tumours [75].

#### Pain

Is an important possible confounder when assessing the association between opioid exposure and cancer-related outcomes, which is rarely adequately measured and adjusted. This is illustrated in the findings from Elkholm et al. (2014) and Houston et al. (2023) discussed above. It is possible that confounding by indication occurs whereby the pain of a pre-cancerous condition or undiagnosed cancer is what prompts opioid prescription. It is also possible that pain directly increases the risk of developing cancer [76–78]; however, it is important to note that these studies did not evaluate opioid use. This hypothesis of a role for pain is supported by animal data in which pain significantly attenuated anti-cancer immunity and increased tumour take in animal inoculated with cancer cells [79–81]. Studies that show an increased cancer risk in participants exposed to the non-opioid analgesic paracetamol [30], or among the control group of non-opioid users who had pain, compared to the reference group of non-opioid users without pain, further supports the idea that pain may contribute to the observed association between opioid use and increased cancer risk [52]. Finally, pain could be an indication of having an underlying chronic health condition that in itself increases cancer risk, such as inflammatory bowel diseases, pancreatitis, or gastroesophageal reflux disease. Only two of studies evaluated in this review had adjusted for use of other analgesics, a proxy for pain [44, 46] and one adjusted for underlying musculoskeletal condition [44].

### Confounders for decreased cancer risk

Five of the studies reporting organ-specific risk of cancer associated with opioid use demonstrate a decreased breast cancer risk [29, 33, 35, 43, 50], with SMR and SIR ranging from 0.36 to 0.6, and the OR being at 0.96 in one case–control study [29]. In contrast Sun et al. found an absolute HR of 3.23 (2.27–4.35) in patients receiving prescription opioids for pain *vs* non opioid users [49]. It has been proposed that early onset of pregnancy may contribute to lower risk of breast cancer in recreational opioid users [33], and opioid-dependent women tend to have an earlier onset of pregnancy when compared to non-opioid dependent women [82]. Epidemiological studies have established a strong and life-long breast cancer protective effect of early full-term pregnancy in humans [83–85]. This data has been confirmed and explained in rodent models [86–88]. A second possible explanation for lower risk of breast cancer in opioid users may relate to higher rates of screening in this population. Indeed, compared with women not prescribed opioids, women receiving opioids are reportedly more likely to visit their doctor and have greater adjusted odds of receiving breast, cervical, and colorectal cancer screening [89].

### Organ-specificity of the effect of opioids on cancer

There is growing evidence that the opioids may affect the risk of cancer in an organ-specific fashion. In their recent monograph, the International Agency for Research on Cancer (IARC) of the World Health Organisation (WHO) listed opium consumption as carcinogenic to humans, with sufficient evidence for cancers of the lung, larynx and bladder and limited evidence for cancers of the oesophagus, stomach, pancreas, and pharynx, but no evidence of an effect on cancer risk in other organs [26, 90]. It is unclear whether the carcinogenic effect of opium relies on the opioid alkaloids it contains[91], but emerging reports indicate a similar effect for pharmaceutical opioid molecules [5, 6]. The results from our systematic review are consistent with the possibility that opioids may increase the risk for lung, pancreatic, and liver cancers, while decreasing the risk for breast and to a lesser extent colon cancer – although this remains to be demonstrated by studies that address the confounders associated with the populations that are studied.

### Mechanisms of action of opioids in cancer

The literature on the genotoxicity of opioids in *in vitro* assays is discrepant [92–94] and published data suggest opioids are not genotoxic *in vivo* [95, 96], although when administered to rodents some of them (morphine, heroin) result in chromosome damage that requires the presence of adrenal corticosteroids [97, 98]. Opioids exert a plethora of effects on different physiological and cellular functions, involving opioid as well as non-opioid receptors, such as toll-like receptor 4 (TLR4) or the epidermal growth factor receptor (EGFR) [99–101]. Both positive and negative actions (as well as no effect) have been shown *in vitro*, *ex vivo* and *in vivo* (reviewed in [102, 103]). Opioids directly affect tumour cell proliferation and apoptosis with an outcome that seems largely dependent on the concentrations used [104, 105] and they modulate cellular adhesion, migration and invasion [106–108]. Opioids induce changes to key cellular metabolic pathways important in cancer, namely glycolysis, the tricarboxylic acid cycle, glutaminolysis, and oxidative phosphorylation. Notably, opioids modulate energy production by increasing intracellular glucose levels and production of lactic acid, and reducing ATP levels. They further modulate redox balance, potentially allowing cancer cells to overcome reactive oxygen species-mediated apoptosis [109]. They indirectly affect tumour growth and metastasis *via* modulation of anticancer immunity, e.g. depressing the activity of natural killer cells, in a drug-specific fashion [110] or toning down the pro-invasive interaction between cancer cells and macrophages [111]. The effect of opioids on tumour angiogenesis in animal models is equally controversial [13, 112,114,113. ]. It is likely that opioids exert both pro-and anti-cancer actions that may vary between molecules and with experimental conditions, especially concentrations and doses, duration of exposure and the presence of pain for animal models. This underlines the importance of evaluating the available epidemiological data to assess what the overall effect of opioids might be in humans.

## Conclusions

This review synthesizes the available evidence on the relationship between opioid use and the risks of cancer incidence and mortality, highlighting both suggestive findings and critical limitations. While a small increase in cancer risk is possible, the reliability of existing studies is constrained by substantial methodological challenges. Most studies relied on national insurance or registry databases that were not developed for research purposes and lacked data on major confounders. Additionally, many studies used standardized comparisons to the general population rather than robust designs capable of addressing key biases. Confounding factors such as smoking, alcohol use, socioeconomic status, chronic pain, and comorbidities, as well as confounding by indication and reverse causation, were neither properly considered nor adequately addressed in most available studies, significantly limiting the reliability of the evidence. Moreover, current research lacks granular data on opioid characteristics, such as type, administration route, receptor targets, dosage, and duration of use—critical details for clarifying potential mechanisms of any association with cancer risk. The single-timepoint assessment of opioid use (i.e., only at the time of recruitment) is another limitation, as opioid use is often dynamic, and continuous assessment during follow-up would more accurately capture these associations.

Since a randomized trial to evaluate the potential carcinogenicity of pharmaceutical opioids would be neither feasible nor ethical, the highest level of evidence on this topic will necessarily come from high-quality epidemiological studies combined with triangulation through other methods. Given the rapidly increasing prevalence of opioid use worldwide, conducting rigorous epidemiological studies with repeated measures of opioid exposure, validation by exposure biomarkers, larger sample sizes, and methods to minimize bias from confounding and reverse causation is an urgent priority.

## Supplementary Information

Below is the link to the electronic supplementary material.

Supplementary file1 (DOCX 41 KB)

Supplementary file2 (DOCX 58 KB)

Supplementary file3 (DOCX 50 KB)

Supplementary file4 (DOCX 54 KB)

Supplementary file5 (DOCX 52 KB)

## Data Availability

All data is available in the manuscript and associated online resources.
