# Supplementary material for: Opioid use and the risk of cancer incidence and mortality: a systematic review
Source: Cancer Metastasis Rev. 2025 Jun 11;44(2):54. doi: 10.1007/s10555-025-10268-0 (PMC12159095; doi:10.1007/s10555-025-10268-0)
Supplement: Supplementary file 1 — Supplementary file1 (DOCX 41 KB) [file 10555_2025_10268_MOESM1_ESM.docx]

**Appendix 1: Search strategy**

**1. Pubmed:** (((((Opioid-Related Disorders[MeSH Terms]) OR (addict OR addicts OR addiction OR user OR users)) OR ((chronic pain[Title/Abstract]) OR (chronic[Title/Abstract] AND pain[Title/Abstract]) OR (non[Title/Abstract] AND cancer[Title/Abstract] AND pain[Title/Abstract]) OR (cancer[Title/Abstract] AND free[Title/Abstract] AND pain[Title/Abstract])OR (chronic pain[MeSH Terms]))) AND ((analgesics, opioid[MeSH Terms]) OR alfentanil[Title/Abstract] OR buprenorphine[Title/Abstract] OR fentanyl[Title/Abstract] OR heroin[Title/Abstract] OR hydromorphone[Title/Abstract] OR methadone[Title/Abstract] OR morphine[Title/Abstract] OR codeine[Title/Abstract] OR oxycodone[Title/Abstract] OR pethidine[Title/Abstract] OR remifentanil[Title/Abstract] OR tapentadol[Title/Abstract] OR tramadol[Title/Abstract])) AND ((mortality[MeSH Terms]) OR (neoplasms[MeSH Terms]) OR mortality[Title/Abstract] OR death[Title/Abstract] OR cancer[Title/Abstract] OR tumour[Title/Abstract] OR malignancy[Title/Abstract] OR malignant[Title/Abstract] OR neoplasm[Title/Abstract] OR neoplasms[Title/Abstract])) NOT (animals [mh] NOT humans [mh])

**2. EMBASE:** ('opiate'/exp OR alfentanil:ti,ab OR buprenorphine:ti,ab OR fentanyl:ti,ab OR heroin:ti,ab OR hydromorphone:ti,ab OR methadone:ti,ab OR morphine:ti,ab OR codeine:ti,ab OR oxycodone:ti,ab OR pethidine:ti,ab OR remifentanil:ti,ab OR tapentadol:ti,ab OR tramadol:ti,ab) AND ('mortality'/exp OR 'neoplasm'/exp OR mortality:ti,ab OR death:ti,ab OR cancer:ti,ab OR tumour:ti,ab OR malignancy:ti,ab OR malignant:ti,ab OR neoplasm:ti,ab OR neoplasms:ti,ab) AND ('narcotic dependence'/exp OR 'drug:ti,ab and dependence:ti,ab' OR addict:ti,ab OR addiction:ti,ab OR user:ti,ab OR users:ti,ab OR 'chronic pain'/exp OR (chronic:ti,ab AND pain:ti,ab) OR (non:ti,ab AND cancer:ti,ab AND pain:ti,ab) OR (cancer:ti,ab AND free:ti,ab AND pain:ti,ab)) NOT ('animals'/exp NOT 'humans'/exp)

**3. Web of Science:** (((AB=(addict OR addicts OR addiction OR user OR users OR dependant OR depend)) OR (TI=(addict OR addicts OR addiction OR user OR users OR dependant OR depend))) OR AB=(chronic pain OR (non AND cancer AND pain) OR (cancer AND free AND pain))) OR TI=(chronic pain OR (non AND cancer AND pain) OR (cancer AND free AND pain)) *AND* (AB=(opioid OR opioids OR opiate OR opiates OR alfentanil OR buprenorphine OR fentanyl OR heroin OR hydromorphone OR methadone OR morphine OR codeine OR oxycodone OR pethidine OR remifentanil OR tapentadol OR tramadol)) OR TI=(opioid OR opioids OR opiate OR opiates OR alfentanil OR buprenorphine OR fentanyl OR heroin OR hydromorphone OR methadone OR morphine OR codeine OR oxycodone OR pethidine OR remifentanil OR tapentadol OR tramadol) *AND* (AB=(mortality OR neoplasm OR death OR cancer OR tumour OR malignancy OR malignant OR neoplasms)) OR TI=(mortality OR neoplasm OR death OR cancer OR tumour OR malignancy OR malignant OR neoplasms)

**4. PsycInfo:** ((MA opioid-related disorders) OR **(**TI ( opioid addiction or opioid dependent or opioid use disorder ) OR ( addict OR addicts OR addiction OR user OR users ) OR AB ( opioid addiction or opioid dependent or opioid use disorder ) OR ( addict OR addicts OR addiction OR user OR users)) OR MA chronic pain OR ( TI ( chronic pain OR non cancer pain OR cancer free pain ) OR AB ( chronic pain OR non cancer pain OR cancer free pain ))) *AND* ((MA, Analgesics, Opioids) OR (TI ( opioid OR opioids OR opiate OR opiates OR alfentanil OR buprenorphine OR fentanyl OR heroin OR hydromorphone OR methadone OR morphine OR codeine OR oxycodone OR pethidine OR remifentanil OR tapentadol OR tramadol ) OR AB ( opioid OR opioids OR opiate OR opiates OR alfentanil OR buprenorphine OR fentanyl OR heroin OR hydromorphone OR methadone OR morphine OR codeine OR oxycodone OR pethidine OR remifentanil OR tapentadol OR tramadol ))) *AND* ((MA mortality OR MA neoplasms) OR (TI (mortality OR neoplasm OR death OR cancer OR tumour OR malignancy OR malignant OR neoplasms ) OR AB (mortality OR neoplasm OR death OR cancer OR tumour OR malignancy OR malignant OR neoplasms)))

**5. IPA:** ( (TI ( opioid addiction or opioid dependent or opioid use disorder ) OR ( addict OR addicts OR addiction OR user OR users ) OR AB ( opioid addiction or opioid dependent or opioid use disorder ) OR ( addict OR addicts OR addiction OR user OR users)) OR MA chronic pain OR ( TI ( chronic pain OR non cancer pain OR cancer free pain ) OR AB ( chronic pain OR non cancer pain OR cancer free pain )) ) AND ( (TI ( opioid OR opioids OR opiate OR opiates OR alfentanil OR buprenorphine OR fentanyl OR heroin OR hydromorphone OR methadone OR morphine OR codeine OR oxycodone OR pethidine OR remifentanil OR tapentadol OR tramadol ) OR AB ( opioid OR opioids OR opiate OR opiates OR alfentanil OR buprenorphine OR fentanyl OR heroin OR hydromorphone OR methadone OR morphine OR codeine OR oxycodone OR pethidine OR remifentanil OR tapentadol OR tramadol )) ) AND ( (TI (mortality OR neoplasm OR death OR cancer OR tumour OR malignancy OR malignant OR neoplasms ) OR AB (mortality OR neoplasm OR death OR cancer OR tumour OR malignancy OR malignant OR neoplasms)) )

**6. CINAHL:** ((TI ( opioid addiction or opioid dependent or opioid use disorder ) OR ( addict OR addicts OR addiction OR user OR users ) OR AB ( opioid addiction or opioid dependent or opioid use disorder ) OR ( addict OR addicts OR addiction OR user OR users)) OR ( TI ( chronic pain OR non cancer pain OR cancer free pain ) OR AB ( chronic pain OR non cancer pain OR cancer free pain )) ) *AND* ( (TI ( opioid OR opioids OR opiate OR opiates OR alfentanil OR buprenorphine OR fentanyl OR heroin OR hydromorphone OR methadone OR morphine OR codeine OR oxycodone OR pethidine OR remifentanil OR tapentadol OR tramadol ) OR AB ( opioid OR opioids OR opiate OR opiates OR alfentanil OR buprenorphine OR fentanyl OR heroin OR hydromorphone OR methadone OR morphine OR codeine OR oxycodone OR pethidine OR remifentanil OR tapentadol OR tramadol )) ) *AND* ( (TI (mortality OR neoplasm OR death OR cancer OR tumour OR malignancy OR malignant OR neoplasms ) OR AB (mortality OR neoplasm OR death OR cancer OR tumour OR malignancy OR malignant OR neoplasms)) )

**7. Scopus:** (TITLE-ABS (&apos;opioid addiction&apos; or &apos;opioid dependent&apos; or &apos;opioid use disorder&apos;) OR ( addict OR addicts OR addiction OR user OR users)) OR (TITLE-ABS (chronic pain OR non cancer pain OR cancer free pain)) AND (TITLE-ABS (opioid OR opioids OR opiate OR opiates OR alfentanil OR buprenorphine OR fentanyl OR heroin OR hydromorphone OR methadone OR morphine OR codeine OR oxycodone OR pethidine OR remifentanil OR tapentadol OR tramadol)) AND (TITLE-ABS (mortality OR neoplasm OR death OR cancer OR tumour OR malignancy OR malignant OR neoplasms))

**Appendix 2: Excluded studies and reasons for exclusion**

| **Study** | **Reason for exclusion (n=148)** |
| --- | --- |
| [1-4] | Studies focused on opium (n=4) |
| [5, 6] | Studies focused on Heroin (n=2) |
| [7-19] | Review articles (n=13) |
| [20, 21] | Commentary (n=2) |
| [22, 23] | Corrigendum (n=2) |
| [24-65] | Conference abstract (n=42) |
| [66-74] | Study population had cancer prior to exposure (n=9) |
| [75-142] | Opioid exposure and outcome association not descried (n=68) |
| [143-147] | No comparator (n=5) |

**References**

1. Ghadimi T, Gheitasi B, Nili S, Karimi M, Ghaderi E. Occupation, smoking, opium, and bladder cancer: A case-control study. South Asian Journal of Cancer. 2015;4(3):111-4. doi: 10.4103/2278-330X.173174.

2. Ilic I, Ilic M. Opium consumption and pancreatic cancer: A meta-analysis. Cancer Epidemiology. 2022;81. doi: 10.1016/j.canep.2022.102287.

3. Nalini M, Khoshnia M, Kamangar F, Sharafkhah M, Poustchi H, Pourshams A, et al. Joint effect of diabetes and opiate use on all-cause and cause-specific mortality: the Golestan cohort study. Int J Epidemiol. 2021;50(1):314-24. doi: 10.1093/ije/dyaa126.

4. Vazirinejad R, Najafipour R, Rezaeian M, Ghazizadeh A, Mohammadi FD. Opium and risk of gastrointestinal cancer: A case-control study. Turkish Journal of Medical Sciences. 2020;50(4):697-705. doi: 10.3906/sag-1907-100.

5. Carli M, Fini E, De Luca G, Scarselli M, Lamanna F, Vico T, et al. Methadone dose escalation in patients with opioid use disorder and cancer as a strategy for controlling cancer-related pain: A case series. Palliat Support Care. 2023:1-4. doi: 10.1017/S1478951523001293.

6. Pavarin RM, Fioritti A, Sanchini S. Mortality trends among heroin users treated between 1975 and 2013 in Northern Italy: Results of a longitudinal study. J Subst Abuse Treat. 2017;77:166-73. doi: 10.1016/j.jsat.2017.02.009.

7. Chou R, Deyo R, Devine B, Hansen R, Sullivan S, Jarvik JG, et al. The Effectiveness and Risks of Long-Term Opioid Treatment of Chronic Pain. Evid Rep Technol Assess (Full Rep). 2014(218):1-219. doi: 10.23970/AHRQEPCERTA218.

8. Degenhardt L, Bucello C, Mathers B, Briegleb C, Ali H, Hickman M, et al. Mortality among regular or dependent users of heroin and other opioids: a systematic review and meta-analysis of cohort studies. Addiction. 2011;106(1):32-51. doi: 10.1111/j.1360-0443.2010.03140.x.

9. Els C, Jackson TD, Kunyk D, Lappi VG, Sonnenberg B, Hagtvedt R, et al. Adverse events associated with medium- and long-term use of opioids for chronic non-cancer pain: an overview of Cochrane Reviews. Cochrane Database Syst Rev. 2017;10(10):CD012509. doi: 10.1002/14651858.CD012509.pub2.

10. Giorgetti A, Pascali J, Montisci M, Amico I, Bonvicini B, Fais P, et al. The role of risk or contributory death factors in methadone-related fatalities: A review and pooled analysis. Metabolites. 2021;11(3). doi: 10.3390/metabo11030189.

11. Hser YI, Evans E, Grella C, Ling W, Anglin D. Long-term course of opioid addiction. Harv Rev Psychiatry. 2015;23(2):76-89. doi: 10.1097/HRP.0000000000000052.

12. Hulse GK, English DR, Milne E, Holman CDJ. The quantification of mortality resulting from the regular use of illicit opiates. Addiction. 1999;94(2):221-9. doi: 10.1046/j.1360-0443.1999.9422216.x.

13. Long B, Gottlieb M. Is Opioid Agonist Treatment Associated With Reduced Risk of Overall and Cause-Specific Mortality in Opioid-Dependent People? Annals of Emergency Medicine. 2021;78(6):776-8.

14. Phua SW, Chen LC, Harvey J. A systematic review of the effectiveness outcomes and adverse events associated with long-term opioid therapy for patients with chronic non-cancer pain. International Journal of Pharmacy Practice. 2015;23:61-2. doi: 10.1111/ijpp.12213.

15. Plein LM, Rittner HL. Opioids and the immune system - friend or foe. Br J Pharmacol. 2018;175(14):2717-25. doi: 10.1111/bph.13750.

16. Santo T, Jr., Clark B, Hickman M, Grebely J, Campbell G, Sordo L, et al. 'Association of opioid agonist treatment with all-cause mortality and specific causes of death among people with opioid dependence: A systematic review and meta-analysis': Correction. JAMA Psychiatry. 2021;78(9):1044-.

17. Schottenfeld D. Cancer risks of medical treatment. CA: A Cancer Journal for Clinicians. 1982;32(5):258-79. doi: 10.3322/canjclin.32.5.258.

18. Sheikh M, Brennan P, Mariosa D, Robbins HA. Opioid medications: an emerging cancer risk factor? Br J Anaesth. 2023;130(3):e401-e3. doi: 10.1016/j.bja.2022.12.007.

19. Tölle T, Fitzcharles MA, Häuser W. Is opioid therapy for chronic non-cancer pain associated with a greater risk of all-cause mortality compared to non-opioid analgesics? A systematic review of propensity score matched observational studies. Eur J Pain. 2021;25(6):1195-208. doi: 10.1002/ejp.1742.

20. Chu WM, Huang PS, Wei JC. Association between long-term opioid use and cancer risk in patients with chronic pain. Comment on Br J Anaesth 2022; 129: 84-91. Br J Anaesth. 2022;129(4):e104-e5. doi: 10.1016/j.bja.2022.07.007.

21. Wu SY, Huang JY, Lai YR, Lin JA. Association between long-term opioid use and cancer risk in patients with chronic pain. Response to Br J Anaesth 2022; 129: e104-5. Br J Anaesth. 2023;130(6):e488-e9. doi: 10.1016/j.bja.2023.03.002.

22. Pierce M, Bird SM, Hickman M, Millar T. 'National record linkage study of mortality for a large cohort of opioid users ascertained by drug treatment or criminal justice sources in England, 2005–2009': Corrigendum. Drug and Alcohol Dependence. 2015;156:315-. doi: 10.1016/j.drugalcdep.2015.09.016.

23. Rosca P, Haklai Z, Goldberger N, Peres Z, Margolis A, Ponizovsky AM. Corrigendum to "Mortality and causes of death among users of methadone maintenance treatment in Israel, 1999-2008" [Drug Alcohol Depend. 125 (2012) 160-163]. Drug and Alcohol Dependence. 2012;126(1-2):277. doi: 10.1016/j.drugalcdep.2012.09.001.

24. Allen C, Meeraus W, Donegan K. Comparative risk of all-cause mortality in older patients prescribed codeine or tramadol for non-malignant pain: Retrospective cohort study. Pharmacoepidemiology and Drug Safety. 2016;25:368. doi: 10.1002/pds.4070.

25. Allen C, Meeraus W, Donegan K. Comparative risk of cause-specific mortality in older patients prescribed codeine or tramadol for non-malignant pain: Retrospective cohort study. Drug Safety. 2017;40(10):993-4. doi: 10.1007/s40264-017-0580-8.

26. As-Sanie S, Soliman AM, Evans K, Erpelding N, Lanier R, Katz N. PIH48 A retrospective database analysis of short-acting and long-acting opioid use in women with endometriosis: II-patterns of utilization. Value in Health. 2019;22:S190. doi: 10.1016/j.jval.2019.04.848.

27. Benitez-Aurioles J, Jenkins D, Huang YT, Medina CR, Peek N, Jani M. Development and evaluation of machine learning algorithms for the prediction of opioid-related deaths among UK patients with non-cancer pain. Rheumatology (United Kingdom). 2023;62:ii42-ii3. doi: 10.1093/rheumatology/kead104.086.

28. Brookhart MA, Reams D, Butler A, Assimon M, Flythe J, Kshirsagar A. Prescription opioid use and mortality risk in hemodialysis patients. Pharmacoepidemiology and Drug Safety. 2015;24:356. doi: 10.1002/pds.3838.

29. Chang KC, Chen CY, Wang JD, Lu TH. Estimation of life expectancy and the expected years of life loss among heroin users in the era of opiate substitution treatment in Taiwan. Drug and Alcohol Dependence. 2015;156:e41. doi: 10.1016/j.drugalcdep.2015.07.1029.

30. Chang KC, Saxon A, Woody GE, Wang JD, Hser YI. Causes of death and expected years of life lost among opioid-dependent individuals using agonist therapy in the U.S. and Taiwan. Drug and Alcohol Dependence. 2017;171:e38-e9. doi: 10.1016/j.drugalcdep.2016.08.118.

31. Chou R, Ray W. Long-acting opioids for chronic noncancer pain were linked to mortality. ACP Journal Club. 2016;165(6):9-. doi: 10.7326/ACPJC-2016-165-6-034.

32. Chung CP, Callahan ST, Cooper W, Dupont W, Murray K, Hall K, et al. Opioid prescriptions and adverse reactions in children and adolescents without serious diseases. Arthritis and Rheumatology. 2016;68:471-2. doi: 10.1002/art.39977.

33. Chung CP, Dupont WD, Murray KT, Hall K, Michael Stein C, Ray WA. Comparative safety of long-acting opioids for non-cancer pain. Pharmacoepidemiology and Drug Safety. 2016;25:581-2. doi: 10.1002/pds.4070.

34. Dasu NR, Khalid Y, Suga H, Foster CJ, Blair B. Outcomes of patients with opioid use disorder and concomitant chronic pancreatitis vs chronic pancreatitis: A model to predict mortality and hospital length of stay. American Journal of Gastroenterology. 2021;116(SUPPL):S1324-S5. doi: 10.14309/01.ajg.0000786396.26204.0a.

35. Ekholm O, Kurita GP, Højsted J, Juel K, Sjøgren P. Associations between opioid use and mortality in the Danish population. Palliative Medicine. 2014;28(6):814. doi: 10.1177/0269216314532748.

36. Fareed A, Casarella J, Amar R, Vayalapalli S, Drexler K. Premature death in older opiate addicts in methadone maintenance treatment and medical co-morbidities as risk factors. American Journal on Addictions. 2009;18(4):322-3. doi: 10.1080/10550490902928197.

37. George J, Radhakrishnan M, Vichitkulwongsa K, Manghnani P, Suarez-Ramos K. Use of strong opioids for chronic non-cancer pain-a retrospective analysis of practice at pain management centre, Singapore general hospital. Proceedings of Singapore Healthcare. 2011;20:137. doi: 10.1177/20101058110200S101.

38. Graf S, Brunner N, Falcato L, Bruggmann P. Causes of death among patients in opioid maintenance treatment and association with HCV infections: A retrospective analysis. Suchtmedizin in Forschung und Praxis. 2013;15(4):254.

39. Hamina A, Taipale H, Karttunen N, Tolppanen AM, Tanskanen A, Tiihonen J, et al. Hospital-treated pneumonia associated with opioid use among community dwellers with Alzheimer's disease: A nationwide matched retrospective cohort study. European Geriatric Medicine. 2018;9:S240. doi: 10.1007/s41999-018-0097-4.

40. Indupuru HKR, Fraher C, Bambhroliya AB, Meyer E, Meeks JR, Vahidy F. Nationwide estimates of opioid abuse in young stroke patients. Stroke. 2018;49.

41. James J, Jackson SL, Klein JW, Merrill JO, McKinney C, Scott J, et al. Patient characteristics and outcomes following discontinuation of primary care based chronic opioid therapy: A retrospective cohort study. Journal of General Internal Medicine. 2018;33(2):281.

42. Kaplovitch E, Gomes T, Camacho X, Dhalla I, Mamdani MM, Juurlink DN. Gender, dose escalation and mortality during opioid therapy. Clinical Toxicology. 2013;51(4):334. doi: 10.3109/15563650.2013.785188.

43. Khodneva Y, Muntner P, Kertesz S, Howard G, Safford M. Prescription opioid use is associated with increased mortality in the reasons for geographic and racial differences in stroke study. Drug and Alcohol Dependence. 2015;146:e157. doi: 10.1016/j.drugalcdep.2014.09.345.

44. Kim M, Suh HS. Long-Term Tramadol Use and One-Year Mortality in Patients with Chronic Kidney Disease in South Korea. Value in Health. 2023;26(6):S21. doi: 10.1016/j.jval.2023.03.112.

45. Lavignasse P, Lowenstein W, Batel P, Constant MV, Jourdain JJ, Kopp P, et al. Economic and social effects of high-dose buprenorphine substitution therapy. Six-month results. Ann Med Interne (Paris). 2002;153(3 Suppl):1S20-6.

46. Le T, Park S, Choi M, Simoni-Wastila L. Concomitant sedative and opioid use: Risk of death in medicare beneficiaries with chronic obstructive pulmonary disease. Consultant Pharmacist. 2018;33(10):589.

47. Le TT, Park S, Choi M, Simoni-Wastila L. Concomitant sedative and opioid use:A dose response analysis. Pharmacoepidemiology and Drug Safety. 2018;27:292. doi: 10.1002/pds.4629.

48. Lee AF, Lee CC. 316 Use of Opioids and Outcomes of Pneumonia: Results from the United States Nationwide Inpatient Sample. Annals of Emergency Medicine. 2019;74(4):S124. doi: 10.1016/j.annemergmed.2019.08.275.

49. Lee YH, Huang YN, Chen HY. The mortality and medical service utilization by long-term opioids patients for chronic non-cancer pain. Pharmacotherapy. 2017;37(12):e196. doi: 10.1002/phar.2052.

50. Li L, Setoguchi S, Cabral H, Jick S. Opioids and risk of type 2 diabetes in adults with non-cancer pain. Pain Physician. 2013;16(1):77-88.

51. Lorenzini KI, Wainstein L, Bovet L, Ramlawi M, Khan R, Spechbach H, et al. The epidemic of opioid misuse, a threat for Switzerland? A pilot study at the Geneva University Hospitals. Drug Safety. 2018;41(11):1214-5. doi: 10.1007/s40264-018-0719-2.

52. Ma J, Wang RJ, Su MF, Liu MX, Shi J, Lu L, et al. Effects of opiate substitution treatment on mortality for opioids users: A systematic review and meta-analysis. The Lancet. 2017;390(SPEC.ISS 1):56.

53. Madi MY, Alsakarneh S, Kilani Y, Al-Smadi D, Farooq U, Rocca AC, et al. Increased mortality among hospitalized patients with chronic pancreatitis and opioid use disorder: a nationwide inpatient analysis. Gastroenterology. 2024;166(5):S-744. doi: 10.1016/S0016-5085(24)02193-0.

54. Potukuchi PK, Sumida K, Spivey CA, Dashputre AA, Gaipov A, Kar S, et al. Association between pre-ESRD opioid use and post-ESRD mortality. Journal of the American Society of Nephrology. 2019;30:996.

55. Rege S, Ames S, Frey A, Holstege C. Characterizing the opioid-related mortality in the United States using a national poison database. Clinical Toxicology. 2020;58(11):1206. doi: 10.1080/15563650.2020.1804238.

56. Rhee JY, Sager ZS, Brizzi K. Buprenorphine Low-Dose Initiation to Decrease Total Opioid Use in Patients with Cancer and Non-Cancer-Related Pain. Journal of Pain and Symptom Management. 2024;67(5):e734-e5. doi: 10.1016/j.jpainsymman.2024.02.225.

57. Risser D, Honigschnabl S, Stichenwirth M, Pfudl S, Sebald D, Kaff A, et al. Mortality of opiate users. Forensic Science International. 2003;136:318-.

58. Saunders N, Amartey A, Chiu M, Zhou L, Harron K, Gilbert R, et al. Mortality of mothers of infants born with neonatal abstinence syndrome: A population-based twenty year cohort study. Paediatrics and Child Health (Canada). 2017;22:e32. doi: 10.1093/pch/pxx086.082.

59. Shaikh A, Kaye AJ, Meyers S, Le A, Ahlawat S. Pre-existing opioid use worsens outcomes in patients with diverticulitis. Gastroenterology. 2022;162(7):S-517. doi: 10.1016/S0016-5085(22)61228-9.

60. Sheikh M, Alcala K, Mariosa D, Feng X, Sarich P, Weber M, et al. MA03.08 Regular Use of Pharmaceutical Opioids and Subsequent Risk of Lung Cancer. Journal of Thoracic Oncology. 2023;18(11):S107-S8. doi: 10.1016/j.jtho.2023.09.132.

61. Sjøgren P. Population-based studies on chronic pain: The role of opioids. Scandinavian Journal of Pain. 2012;3(3):181. doi: 10.1016/j.sjpain.2012.05.021.

62. Sjøgren P, Grønbæk M, Peuckmann V, Ekholm O. Critical issues on opioids in chronic non-cancer pain: A cohort study. European Journal of Pain. 2009;13:S89. doi: 10.1016/S1090-3801(09)60290-1.

63. Surbhi S, Mallisetty Y, Shrestha P, Sumida K, Thomas F, Kalantar-Zadeh K, et al. Comparative Effectiveness of Opioids vs. Non-Opioid Analgesics on the Risk of ESRD and Mortality Among US Veterans with CKD and Chronic Pain. Journal of the American Society of Nephrology. 2023;34:393.

64. Vandenbossche J, Richarz U, Richards H. A repeat-dose, steady-state pharmacokinetic evaluation of once-daily hydromorphone ER (OROS hydromorphone) in patients with chronic cancer or noncancer pain. Journal of Pain. 2011;12(4):P60. doi: 10.1016/j.jpain.2011.02.243.

65. Watson CPN. Opioids for chronic neuropathic pain: Long-term efficacy and safety issues. Acta Anaesthesiologica Scandinavica. 2009;53((Watson C.P.N.) Faculty of Medicine, University of Toronto, 1 Sir Williams Lane, Toronto, ON, Canada):32. doi: 10.1111/j.1399-6576.2009.02001.x.

66. Boudreau DM, Chen L, Yu O, Bowles EJA, Chubak J. Risk of second breast cancer events with chronic opioid use in breast cancer survivors. Pharmacoepidemiol Drug Saf. 2019;28(5):740-53. doi: 10.1002/pds.4779.

67. Cronin-Fenton DP, Heide-Jørgensen U, Ahern TP, Lash TL, Christiansen PM, Ejlertsen B, et al. Opioids and breast cancer recurrence: A Danish population-based cohort study. Cancer. 2015;121(19):3507-14. doi: 10.1002/cncr.29532.

68. Du W, Chong S, McLachlan AJ, Luo L, Glasgow N, Gnjidic D. Adverse drug reactions due to opioid analgesic use in New South Wales, Australia: a spatial-temporal analysis. BMC Pharmacol Toxicol. 2019;20(1):55. doi: 10.1186/s40360-019-0333-7.

69. Durham DD, Strassels SA, Pinsky PF. Opioid use by cancer status and time since diagnosis among older adults enrolled in the Prostate, Lung, Colorectal, and Ovarian screening trial in the United States. Cancer Med. 2021;10(6):2175-87. doi: 10.1002/cam4.3810.

70. Kurteva S, Tamblyn R, Meguerditchian AN. Predictors of frequent emergency department visits among hospitalized cancer patients: a comparative cohort study using integrated clinical and administrative data to improve care delivery. BMC Health Serv Res. 2023;23(1):887. doi: 10.1186/s12913-023-09854-1.

71. Lindestrand AG, Christiansen ML, Jantzen C, van der Mark S, Andersen SE. Opioids in hip fracture patients: an analysis of mortality and post hospital opioid use. Injury. 2015;46(7):1341-5. doi: 10.1016/j.injury.2015.04.016.

72. Mörttinen-Vallius HP, Huhtala HSA, Hartikainen SA, Jämsen ERK. The Association of Long-Term Opioid Use With Health Care and Home Care Service Use Among Aged Home Care Clients. J Am Med Dir Assoc. 2023;24(6):798-803.e1. doi: 10.1016/j.jamda.2022.11.023.

73. Sun M, Chang CL, Lu CY, Zhang J, Wu SY. Effect of opioids on cancer survival in patients with chronic pain: a propensity score-matched population-based cohort study. Br J Anaesth. 2022;128(4):708-17. doi: 10.1016/j.bja.2021.12.051.

74. Woo HT, Jeong SY, Shin A. The association between prescription drugs and colorectal cancer prognosis: a nationwide cohort study using a medication-wide association study. BMC Cancer. 2023;23(1). doi: 10.1186/s12885-023-11105-9.

75. Agnoli A, Jerant A, Franks P. Cancer screening among women prescribed opioids: A national study. Annals of Family Medicine. 2020;18(1):59-65. doi: 10.1370/afm.2489.

76. Agustí A, Pagès E, Cuxart A, Ballarín E, Vidal X, Teixidor J, et al. Exposure to medicines among patients admitted for hip fracture and the case-fatality rate at 1 year: A longitudinal study. European Journal of Clinical Pharmacology. 2012;68(11):1525-31. doi: 10.1007/s00228-012-1273-y.

77. Allen M, Sproule B, MacDougall P, Furlan A, Murphy L, Borg Debono V, et al. Identifying appropriate outcomes to help evaluate the impact of the Canadian Guideline for Safe and Effective Use of Opioids for Non-Cancer Pain. BMC Anesthesiol. 2020;20(1):6. doi: 10.1186/s12871-020-0930-4.

78. Dauri M, Lazzari M, Casali M, Tufaro G, Sabato E, ASabato AF. Long-Term Efficacy of OROS(R) Hydromorphone Combined with Pregabalin for Chronic Non-Cancer Neuropathic Pain. 2014. p. 309.

79. Baberi F, Kavousi A, Mirtorabi D, Parsa N, Mahdavi SA, Nazari SSH. Original article: Assessing substances abuse-induced mortality rates by autopsy methodin Iran. International Journal of Medical Toxicology and Forensic Medicine. 2021;11(2). doi: 10.32598/IJMTFM.V11I2.32400.

80. Bardage C, Grünewald M, Tuvendal P, Ljung R. First opioid prescribing in Sweden: drugs, doses, and diagnoses in more than 600 000 opioid-naïve and cancer free patients. Journal of Substance Use. 2024;29(4):617-23. doi: 10.1080/14659891.2023.2214218.

81. Barrantes F, Luan FL, Kommareddi M, Alazem K, Yaqub T, Roth RS, et al. A history of chronic opioid usage prior to kidney transplantation may be associated with increased mortality risk. Kidney Int. 2013;84(2):390-6. doi: 10.1038/ki.2013.136.

82. Basu D, Mattoo SK, Malhotra A, Gupta N, Malhotra R. A longitudinal study of male buprenorphine addicts attending an addiction clinic in India. Addiction. 2000;95(9):1363-72. doi: 10.1046/j.1360-0443.2000.95913637.x.

83. Bernard JP, Havnes I, Slørdal L, Waal H, Mørland J, Khiabani HZ. Methadone-related deaths in Norway. Forensic Science International. 2013;224(1-3):111-6. doi: 10.1016/j.forsciint.2012.11.010.

84. Blanch B, Pearson SA, Haber PS. An overview of the patterns of prescription opioid use, costs and related harms in Australia. 5 ed2014. p. 1159-66.

85. Braun HJ, Schwab MP, Jin C, Amara D, Mehta NJ, Grace TR, et al. Opioid use prior to liver transplant is associated with increased risk of death after transplant. Am J Surg. 2021;222(1):234-40. doi: 10.1016/j.amjsurg.2020.11.039.

86. Bromley M. The shifting paradigm of pain management among US elderly community dwellers. ProQuest Information & Learning; 2023.

87. Burgstaller JM, Held U, Signorell A, Blozik E, Steurer J, Wertli MM. Increased risk of adverse events in non-cancer patients with chronic and high-dose opioid use-A health insurance claims analysis. PLoS One. 2020;15(9):e0238285. doi: 10.1371/journal.pone.0238285.

88. Chung CP, Callahan ST, Cooper WO, Dupont WD, Murray KT, Franklin AD, et al. Individual short-acting opioids and the risk of opioid-related adverse events in adolescents. Pharmacoepidemiology and Drug Safety. 2019;28(11):1448-56. doi: 10.1002/pds.4872.

89. Chung CP, Dupont WD, Murray KT, Hall K, Stein CM, Ray WA. Comparative out-of-hospital mortality of long-acting opioids prescribed for non-cancer pain: A retrospective cohort study. Pharmacoepidemiol Drug Saf. 2019;28(1):48-53. doi: 10.1002/pds.4619.

90. Corriere MA, Daniel LL, Dickson AL, Nepal P, Hall K, Plummer WD, et al. Concurrent Gabapentin and Opioid Use and Risk of Mortality in Medicare Recipients with Non-Cancer Pain. Clin Pharmacol Ther. 2023. doi: 10.1002/cpt.3019.

91. Daniels B, Tervonen HE, Pearson SA. Identifying incident cancer cases in dispensing claims: A validation study using Australia's Repatriation Pharmaceutical Benefits Scheme (PBS) data. International journal of population data science. 2019;5(1):1152. doi: 10.23889/ijpds.v5i1.1152.

92. Degenhardt L, Randall D, Hall W, Law M, Butler T, Burns L. Mortality among clients of a state-wide opioid pharmacotherapy program over 20 years: risk factors and lives saved. Drug Alcohol Depend. 2009;105(1-2):9-15. doi: 10.1016/j.drugalcdep.2009.05.021.

93. Ericson Ø B, Eide D, Lobmaier P, Clausen T. Mortality risk and causes of death among people who use opioids in a take-home naloxone cohort. Drug Alcohol Depend. 2024;255:111087. doi: 10.1016/j.drugalcdep.2024.111087.

94. Ferrantella A, Huerta CT, Quinn K, Mavarez AC, Quiroz HJ, Thorson CM, et al. Risk factors associated with recent opioid-related hospitalizations in children: a nationwide analysis. Pediatr Surg Int. 2022;38(6):843-51. doi: 10.1007/s00383-022-05088-0.

95. Figueroa-Parra G, Jeffery MM, Dabit JY, Chevet B, Valenzuela-Almada MO, Hocaoglu M, et al. Long-Term Opioid Therapy Among Patients With Systemic Lupus Erythematosus in the Community: A Lupus Midwest Network (LUMEN) Study. J Rheumatol. 2023;50(4):504-11. doi: 10.3899/jrheum.220822.

96. Fischer B, Jones W, Urbanoski K, Skinner R, Rehm J. Correlations between prescription opioid analgesic dispensing levels and related mortality and morbidity in Ontario, Canada, 2005-2011. Drug Alcohol Rev. 2014;33(1):19-26. doi: 10.1111/dar.12089.

97. Fleming JN, Taber DJ, Pilch NA, Mardis CR, Gilbert RE, Wilson LZ, et al. Association of Pretransplantion Opioid Use with Graft Loss or Death in Liver Transplantation Patients with Model of End-Stage Liver Disease Exceptions. J Am Coll Surg. 2018;226(4):651-9. doi: 10.1016/j.jamcollsurg.2017.12.025.

98. Fontenla A, Vaamonde A, Flórez G. Mortality in patients addicted to opioids across 30-year follow-up. Adicciones. 2023:1803. doi: 10.20882/adicciones.1803.

99. Franklin GM, Mai J, Wickizer T, Turner JA, Fulton-Kehoe D, Grant L. Opioid dosing trends and mortality in Washington State workers' compensation, 1996-2002. Am J Ind Med. 2005;48(2):91-9. doi: 10.1002/ajim.20191.

100. Gabrhelík R, Hesse M, Nechanská B, Handal M, Mravčík V, Tjagvad C, et al. Large variations in all-cause and overdose mortality among >13,000 patients in and out of opioid maintenance treatment in different settings: a comparative registry linkage study. Front Public Health. 2023;11:1179763. doi: 10.3389/fpubh.2023.1179763.

101. Geile J, Maas A, Kraemer M, Doberentz E, Madea B. Fatal misuse of transdermal fentanyl patches. Forensic Sci Int. 2019;302:109858. doi: 10.1016/j.forsciint.2019.06.016.

102. Gjersing L, Bretteville-Jensen AL. Patterns of substance use and mortality risk in a cohort of 'hard-to-reach' polysubstance users. Addiction. 2018;113(4):729-39. doi: 10.1111/add.14053.

103. Gokhale MN, Martin B. The temporal association between prescribed opioids and the national death rate due to opioid poisoning. Value in Health. 2011;14(3):A13-A4.

104. Gomes T, Mamdani MM, Dhalla IA, Paterson JM, Juurlink DN. Opioid dose and drug-related mortality in patients with nonmalignant pain. Arch Intern Med. 2011;171(7):686-91. doi: 10.1001/archinternmed.2011.117.

105. Grönbladh L, Gunne L. Methadone-assisted rehabilitation of Swedish heroin addicts. Drug Alcohol Depend. 1989;24(1):31-7. doi: 10.1016/0376-8716(89)90005-7.

106. Grönbladh L, Ohlund LS, Gunne LM. Mortality in heroin addiction: impact of methadone treatment. Acta Psychiatr Scand. 1990;82(3):223-7. doi: 10.1111/j.1600-0447.1990.tb03057.x.

107. Hosbach I, Zenz M. Correlation between intrathecal opioids and gynecomastia - A case report. Schmerz. 2003;17(1):60-2. doi: 10.1007/s00482-002-0171-0.

108. Hser YI, Mooney LJ, Saxon AJ, Miotto K, Bell DS, Zhu YH, et al. High Mortality Among Patients With Opioid Use Disorder in a Large Healthcare System. Journal of Addiction Medicine. 2017;11(4):315-9. doi: 10.1097/ADM.0000000000000312.

109. Hsu G, Kovács B. Association between county level cannabis dispensary counts and opioid related mortality rates in the United States: panel data study. BMJ. 2021;372:m4957. doi: 10.1136/bmj.m4957.

110. Inacio MC, Lang C, Caughey GE, Bray SCE, Harrison SL, Whitehead C, et al. The Registry of Senior Australians outcome monitoring system: Quality and safety indicators for residential aged care. International Journal for Quality in Health Care. 2020;32(8):502-10. doi: 10.1093/intqhc/mzaa078.

111. Jensen MK, Thomsen AB, Højsted J. 10-year follow-up of chronic non-malignant pain patients: opioid use, health related quality of life and health care utilization. Eur J Pain. 2006;10(5):423-33. doi: 10.1016/j.ejpain.2005.06.001.

112. Jiménez-Treviño L, Martínez-Cao C, Sánchez-Lasheras F, Iglesias C, Antuña MJ, Riera L, et al. A 35-year follow-up study of patients admitted to methadone treatment between 1982-1984 in Asturias, Spain. Adicciones. 2023;35(3):303-14. doi: 10.20882/adicciones.1662.

113. Kaplovitch E, Gomes T, Camacho X, Dhalla IA, Mamdani MM, Juurlink DN. Sex Differences in Dose Escalation and Overdose Death during Chronic Opioid Therapy: A Population-Based Cohort Study. PLoS One. 2015;10(8):e0134550. doi: 10.1371/journal.pone.0134550.

114. Kelty E, Chrzanowska A, Preena DB. Fatalities in Patients with Opioid Use Disorders. 2022. p. 1851-67.

115. Kessler ER, Shah M, Gruschkus SK, Raju A. Cost and quality implications of opioid-based postsurgical pain control using administrative claims data from a large health system: opioid-related adverse events and their impact on clinical and economic outcomes. Pharmacotherapy. 2013;33(4):383-91. doi: 10.1002/phar.1223.

116. Kringsholm B. Deaths among drug addicts in Denmark in 1968-1986. Forensic Sci Int. 1988;38(1-2):139-49. doi: 10.1016/0379-0738(88)90017-5.

117. Kringsholm B, Voigt J, Dalgaard JB, Simonsen J. Deaths among narcotic addicts in Denmark in 1978 and 1979. Forensic Sci Int. 1981;18(1):19-30. doi: 10.1016/0379-0738(81)90136-5.

118. Kshirsagar AV, Reams D, Assimon MM, Butler AM, Flythe JE, Brookhart MA. Opioid Use Associates with Infection Related Morbidity and Mortality in Hemodialysis Patients. Journal of the American Society of Nephrology. 2015;26:543A-4A.

119. Lewer D, Jones NR, Hickman M, Nielsen S, Degenhardt L. Life expectancy of people who are dependent on opioids: A cohort study in New South Wales, Australia. J Psychiatr Res. 2020;130:435-40. doi: 10.1016/j.jpsychires.2020.08.013.

120. Lindblad R, Hu L, Oden N, Wakim P, Rosa C, VanVeldhuisen P. Mortality Rates Among Substance Use Disorder Participants in Clinical Trials: Pooled Analysis of Twenty-Two Clinical Trials Within the National Drug Abuse Treatment Clinical Trials Network. Journal of Substance Abuse Treatment. 2016;70:73-80. doi: 10.1016/j.jsat.2016.08.010.

121. Linnet K, Thorsteinsdottir HS, Sigurdsson JA, Sigurdsson EL, Gudmundsson LS. Co-prescribing of opioids and benzodiazepines/Z-drugs associated with all-cause mortality-A population-based longitudinal study in primary care with weak opioids most commonly prescribed. Front Pharmacol. 2022;13:932380. doi: 10.3389/fphar.2022.932380.

122. Mosher HJ, Jiang L, Vaughan Sarrazin MS, Cram P, Kaboli PJ, Vander Weg MW. Prevalence and characteristics of hospitalized adults on chronic opioid therapy. J Hosp Med. 2014;9(2):82-7. doi: 10.1002/jhm.2113.

123. Nosyk B, Min JE, Homayra F, Kurz M, Guerra-Alejos BC, Yan R, et al. Buprenorphine/Naloxone vs Methadone for the Treatment of Opioid Use Disorder. JAMA. 2024. doi: 10.1001/jama.2024.16954.

124. Papadomanolakis-Pakis N, Moore KM, Peng Y, Gomes T. Prescription opioid characteristics at initiation for non-cancer pain and risk of treated opioid use disorder: A population-based study. Drug Alcohol Depend. 2021;221:108601. doi: 10.1016/j.drugalcdep.2021.108601.

125. Peles E, Schreiber S, Adelson M. 15-Year survival and retention of patients in a general hospital-affiliated methadone maintenance treatment (MMT) center in Israel. Drug Alcohol Depend. 2010;107(2-3):141-8. doi: 10.1016/j.drugalcdep.2009.09.013.

126. Randall HB, Alhamad T, Schnitzler MA, Zhang Z, Ford-Glanton S, Axelrod DA, et al. Survival implications of opioid use before and after liver transplantation. Liver Transplantation. 2017;23(3):305-14. doi: 10.1002/lt.24714.

127. Russolillo A, Moniruzzaman A, Somers JM. Association of Methadone Treatment With Substance-Related Hospital Admissions Among a Population in Canada With a History of Criminal Convictions. JAMA Netw Open. 2019;2(3):e190595. doi: 10.1001/jamanetworkopen.2019.0595.

128. Safarudin R, LeMasters T, Khan S, Sambamoorthi U. Prescription Opioid Use before and after Diagnosis of Cancer Among Older Cancer Survivors With Non-Cancer Chronic Pain Conditions (NCPCs): An Application of Group-Based Trajectory Modeling (GBTM). Cancer Control. 2024;31:10732748241290769. doi: 10.1177/10732748241290769.

129. Saitz R, Gaeta J, Cheng DM, Richardson JM, Larson MJ, Samet JH. Risk of mortality during four years after substance detoxification in urban adults. J Urban Health. 2007;84(2):272-82. doi: 10.1007/s11524-006-9149-z.

130. Salkar M, Ramachandran S, Bentley JP, Eriator I, McGwin G, Twyner CC, et al. Do Formulation and Dose of Long-Term Opioid Therapy Contribute to Risk of Adverse Events among Older Adults? J Gen Intern Med. 2022;37(2):367-74. doi: 10.1007/s11606-021-06792-8.

131. Saunders KW, Dunn KM, Merrill JO, Sullivan M, Weisner C, Braden JB, et al. Relationship of opioid use and dosage levels to fractures in older chronic pain patients. J Gen Intern Med. 2010;25(4):310-5. doi: 10.1007/s11606-009-1218-z.

132. Sells SB, Chatham LR, Retka RL. Study of differential death rates and causes of death among 9,276 opiate addicts during 1970-1971. Contemporary Drug Problems (USA). 1972;1(Apr):665-706.

133. Shipton EE, Shipton AJ, Williman JA, Shipton EA. Deaths from Opioid Overdosing: Implications of Coroners' Inquest Reports 2008-2012 and Annual Rise in Opioid Prescription Rates: A Population-Based Cohort Study. Pain Ther. 2017;6(2):203-15. doi: 10.1007/s40122-017-0080-7.

134. Sjøgren P, Grønbæk M, Peuckmann V, Ekholm O. A population-based cohort study on chronic pain: the role of opioids. Clin J Pain. 2010;26(9):763-9. doi: 10.1097/AJP.0b013e3181f15daf.

135. Skeie I, Clausen T, Hjemsæter AJ, Landheim AS, Monsbakken B, Thoresen M, et al. Mortality, Causes of Death, and Predictors of Death among Patients On and Off Opioid Agonist Treatment: Results from a 19-Year Cohort Study. Eur Addict Res. 2022;28(5):358-67. doi: 10.1159/000525694.

136. Solomon DH, Rassen JA, Glynn RJ, Garneau K, Levin R, Lee J, et al. The comparative safety of opioids for nonmalignant pain in older adults. Arch Intern Med. 2010;170(22):1979-86. doi: 10.1001/archinternmed.2010.450.

137. Thepsuwan A, Nimmaanrat S, Thongsuksai P. Risk Factors Associated with Prolonged Postoperative Opioids Use in Non-Cancer Patients Undergoing Spinal Surgery: A Retrospective Study. Journal of Health Science and Medical Research. 2022;40(5):571-84. doi: 10.31584/jhsmr.2022869.

138. Tournebize J, Gibaja V, Frauger E, Authier N, Seyer D, Perri-Plandé J, et al. [French trends in the misuse of Fentanyl: From 2010 to 2015]. Therapie. 2020;75(5):491-502. doi: 10.1016/j.therap.2019.11.002.

139. Vahidy S, Li D, Hirji A, Kapasi A, Weinkauf J, Laing B, et al. Pretransplant Opioid Use and Survival After Lung Transplantation. Transplantation. 2020;104(8):1720-5. doi: 10.1097/TP.0000000000003050.

140. Wang HT, Hill AD, Gomes T, Pinto R, Wijeysundera DN, Scales DC, et al. Trends in opioid use before critical illness among elderly patients in Ontario. J Crit Care. 2020;55:128-33. doi: 10.1016/j.jcrc.2019.10.004.

141. Ward R, Tang YL, Axon RN, Casarella J, Whitfield N, Rauch SAM. Effectiveness of a substance use treatment program for veterans with chronic pain and opioid use disorder. J Subst Abuse Treat. 2022;132:108635. doi: 10.1016/j.jsat.2021.108635.

142. Zenz M, Strumpf M, Tryba M. Long-term oral opioid therapy in patients with chronic nonmalignant pain. J Pain Symptom Manage. 1992;7(2):69-77. doi: 10.1016/0885-3924(92)90116-y.

143. Barlass U, Deshmukh A, Beck T, Bishehsari F. Opioid use as a potential risk factor for pancreatic cancer in the United States: An analysis of state and national level databases. PLoS One. 2021;16(1):e0244285. doi: 10.1371/journal.pone.0244285.

144. Perucci CA, Davoli M, Rapiti E, Abeni DD, Forastiere F. Mortality of intravenous-drug-users in Rome - a cohort study. American Journal of Public Health. 1991;81(10):1307-10. doi: 10.2105/AJPH.81.10.1307.

145. Soyka M, Apelt SM, Lieb M, Wittchen HU. One-year mortality rates of patients receiving methadone and buprenorphine maintenance therapy: a nationally representative cohort study in 2694 patients. J Clin Psychopharmacol. 2006;26(6):657-60. doi: 10.1097/01.jcp.0000245561.99036.49.

146. Soyka M, Träder A, Klotsche J, Backmund M, Bühringer G, Rehm J, et al. Six-year mortality rates of patients in methadone and buprenorphine maintenance therapy: results from a nationally representative cohort study. J Clin Psychopharmacol. 2011;31(5):678-80. doi: 10.1097/JCP.0b013e31822cd446.

147. Vajdic CM, Pour SM, Olivier J, Swart A, O'Connell DL, Falster MO, et al. The impact of blood-borne viruses on cause-specific mortality among opioid dependent people: An Australian population-based cohort study. Drug and Alcohol Dependence. 2015;152:264-71. doi: 10.1016/j.drugalcdep.2015.03.026.
