# Supplementary material for: Opioid use and the risk of cancer incidence and mortality: a systematic review
Source: Cancer Metastasis Rev. 2025 Jun 11;44(2):54. doi: 10.1007/s10555-025-10268-0 (PMC12159095; doi:10.1007/s10555-025-10268-0)
Supplement: Supplementary file 2 — Supplementary file2 (DOCX 58 KB) [file 10555_2025_10268_MOESM2_ESM.docx]

**Supplementary Table S1: New-castle-Ottawa scores for quality assessment of cohort studies**

| **Study** | **Domain 1: Selection** | | | | **Domain 2: Comparability** | **Domain 3: Outcome** | | | **Total score** | **Quality** |
| --- | --- | --- | --- | --- | --- | --- | --- | --- | --- | --- |
|  | *Representativeness of exposed cohort* | *Selection of non-exposed cohort* | *Ascertainment of exposure* | *Demonstration that outcome of interest was not present at start* |  | *Assessment of outcome* | *Follow-up long enough for outcome to occur* | *Adequacy of follow-up of cohorts* |  |  |
| Ekholm (2014) | * | * | * | * | ** | * | * | * | 9 | high |
| Macfarlane (2020) | * | * | * | * | ** | * | * | * | 9 | high |
| Oh (2020) | * | * | * | * | * | * | * | * | 8 | high |
| Song (2022) | * | * | * | * | * | * | * | * | 8 | high |
| Sun (2022) | * | * | * | * | * | * | * | * | 8 | high |
| Zeng (2019) | * | * | * | * | * | * | * | * | 8 | high |
| Grinshpoon (2011) | * | - | * | * | * | * | * | * | 7 | high |
| Kelty (2017) | * | * | * | - | * | * | * | * | 7 | high |
| Lee (2021) | * | - | * | * | * | * | * | * | 7 | high |
| Oh (2019) | * | * | * | - | * | * | * | * | 7 | high |
| Chang (2015) | - | * | * | - | * | * | * | * | 6 | moderate |
| Hser (2019) | - | * | * | - | * | * | * | * | 6 | moderate |
| Kostovksi (2024) | * | - | * | * | - | * | * | * | 6 | moderate |
| Larney (2015) | - | * | * | - | * | * | * | * | 6 | moderate |
| Olfson (2018) | * | - | * | - | * | * | * | * | 6 | moderate |
| Bargagli (2001) | * | - | * | - | - | * | * | * | 5 | moderate |
| Degenhardt (2013) | * | - | * | - | - | * | * | * | 5 | moderate |
| Eide (2023) | * | - | * | - | - | * | * | * | 5 | moderate |
| Gibson (2011) | * | - | * | - | - | * | * | * | 5 | moderate |
| Maxwell (2005) | * | - | * | - | - | * | * | * | 5 | moderate |
| Randall (2011) | * | - | * | - | - | * | * | * | 5 | moderate |
| Rosca (2012) | * | - | * | - | - | * | * | * | 5 | moderate |
| Swart (2012) | * | - | * | - | - | * | * | * | 5 | moderate |
| Veldhuizen (2014) | * | - | * | - | - | * | * | * | 5 | moderate |
| Bjornaas (2008) | - | - | * | - | - | * | * | - | 3 | low |

Note: Total score of 0-3, 4-6 and 7-9 were considered as low, moderate and high quality respectively.
