# Supplementary material for: Opioid use and the risk of cancer incidence and mortality: a systematic review
Source: Cancer Metastasis Rev. 2025 Jun 11;44(2):54. doi: 10.1007/s10555-025-10268-0 (PMC12159095; doi:10.1007/s10555-025-10268-0)
Supplement: Supplementary file 3 — Supplementary file3 (DOCX 50 KB) [file 10555_2025_10268_MOESM3_ESM.docx]

**Supplementary table S2: Newcastle-Ottawa scores for quality assessment of case-control studies**

| **Study** | **Domain 1: Selection** | | | | **Domain 2: Comparability** | **Domain 3: Exposure** | | | **Total score** | **Quality** |
| --- | --- | --- | --- | --- | --- | --- | --- | --- | --- | --- |
|  | *Adequacy of case definition* | *Representative-ness of the cases* | *Selection of controls* | *Definition of controls* |  | *Ascertainment of exposure* | *Same method of ascertainment* | *Non-response rate* |  |  |
| Havidich (2021) | * | * | * | * | ** | * | * | - | 8 | High |
| Houston (2023) | * | * | * | * | ** | * | * | - | 8 | High |

Note: Total score of 0-3, 4-6 and 7-9 were considered as low, moderate and high quality respectively.
