# Supplementary material for: Opioid use and the risk of cancer incidence and mortality: a systematic review
Source: Cancer Metastasis Rev. 2025 Jun 11;44(2):54. doi: 10.1007/s10555-025-10268-0 (PMC12159095; doi:10.1007/s10555-025-10268-0)
Supplement: Supplementary file 4 — Supplementary file4 (DOCX 54 KB) [file 10555_2025_10268_MOESM4_ESM.docx]

**Supplementary table S3: Prevalence of cancer mortality identified from all the included cohort studies**

| **Author (year), design** | **Total participants** | **Total OU** | **cancer mortality in OU, n** | **All-cause mortality in OU, n** | **Comparator** | **% of cancer mortality in OU (of total OU mortality)** | **Prevalence of cancer mortality (% of total OU)** |
| --- | --- | --- | --- | --- | --- | --- | --- |
| Bargagli (2001), Retrospective | 11432 | 11432 | 39 | 1734 | General population | 2.2 | 0.3 |
| Bjornaas (2008), Prospective | 185 | 185 | 3 | 70 | General population | 4.3 | 1.6 |
| Degenhardt (2013), Retrospective | 43789 | 43789 | 212 | 3685 | General population | 5.8 | 0.5 |
| Eide (2023), Prospective | 29486 | 29486 | 523 | 5322 | General population | 9.8 | 1.8 |
| Gibson (2011), Retrospective | 2489 | 2489 | 60 | 478 | General population | 12.6 | 2.4 |
| Kostovksi (2024), Retrospective | 19651 | 19651 | 192 | 2383 | General population | 8.1 | 1.0 |
| Lee (2021), Retrospective | 12990 | 12990 | 13 | 558 | General population | 2.3 | 0.1 |
| Maxwell (2005), Retrospective | 13264 | 13264 | 51 | 766 | General population | 6.7 | 0.4 |
| Olfson (2018), Retrospective | 76325 | 76325 | 536 | 5194 | General population | 10.3 | 0.7 |
| Randall (2011), Retrospective | 43789 | 43789 | 212 | 3533 | General population | 6.0 | 0.5 |
| Rosca (2012), Retrospective | 9818 | 9818 | 96 | 960 | General population | 10.0 | 1.0 |
| Veldhuizen (2014), Retrospective | 68066 | 68066 | 2473 | 13107 | General population | 18.9 | 3.6 |
| Chang (2015), Retrospective | 1283 | 1283 | 15 | 102 | General population | 14.7 | 1.2 |
| Ekholm (2014), Prospective | 13127 | 542 | 32 | 123 | NOU: 335 (2.5%) = NUO1: 85 (*28.1%), NUO2: 250 (*32.5%) | 26.0 | 5.9 |
| Song (2022), Retrospective | 1804019 | 8320 | 371 | 1953 | NOU: 28207 (1.6%) | 19.0 | 4.5 |
| Zeng (2019), Retrospective | 88902 | 61373 | 394 | 1819 | NSAIDs (n=4) and codeine | 21.7 | 0.6 |
| Oh (2019), Retrospective | 822214 | 49429 | 4924 | 20991 | NOU: | 23.5 | 10.0 |
| Larney (2015), Retrospective | 96878 | 60270 | 1823 | 9386 | Old non-OUD: 1240 (*28.1%) | 19.4 | 3.0 |
| Macfarlane (2020), Prospective | 466486 | 25864 | 748 | 1919 | NOU: *7692 (*46.8%) | 39.0 | 2.9 |

**Abbreviations:** NSAIDs, Non-steroidal anti-inflammatory drugs; NOU, Non-opioid users; NOU1, Non-opioid users with chronic pain; NOU2, Non-opioid users without pain; OUD, Opioid use disorder; OU, Opioid users

**Note:**

- Median (q1, q2) cancer mortality % in OU: Overall = 10.0 (6.3, 19.2); studies with general population = 8.1 (0.5, 1.6); studies with NOU = 22.6 (20.0, 25.4)
- Median (q1, q2) prevalence of cancer mortality %: Overall = 1.2 (0.6, 3.0); studies with general population = 1.0 (0.5, 1.6); studies with NOU = 4.0 (2.9, 5.5)
