# Supplementary material for: Opioid use and the risk of cancer incidence and mortality: a systematic review
Source: Cancer Metastasis Rev. 2025 Jun 11;44(2):54. doi: 10.1007/s10555-025-10268-0 (PMC12159095; doi:10.1007/s10555-025-10268-0)
Supplement: Supplementary file 5 — Supplementary file5 (DOCX 52 KB) [file 10555_2025_10268_MOESM5_ESM.docx]

**Supplementary table S4: Cancer incidence identified from all the included cohort studies**

| **Author (year), design** | **Total participants** | **Total OU** | **Cancer incidence, n** | **Comparator** | **Percentage of cancer incidence** |
| --- | --- | --- | --- | --- | --- |
| Kostovski (2024), Retrospective | 19651 | 19651 | 455 | General population | 2.3 |
| Grinshpoon (2011), Retrospective | 18659 | 18659 | 184 | General population | 1.0 |
| Swart (2012), Retrospective | 45412 | 45412 | 819 | General population | 1.8 |
| Ekholm (2014), Prospective | 13127 | 542 | 49 | NOU | 9.0 |
| Oh (2020), Retrospective | 351701 | 25153 | 1871 | NOU | 7.4 |
| Sun (2022), Retrospective | 63610 | 50888 | 4191 | NOU | 8.2 |
| Kelty (2017), Retrospective | 15411 | 5137 | 45 | NOU | 0.9 |
| Oh (2019), Retrospective | 822214 | 49429 | 11737 | NOU | 23.7 |
| Hser (2019), Retrospective | 7728 | 2576 | 363 | NOU | 14.1 |

**Abbreviations:** NOU, Non-opioid users

**Note:** Median (q1, q2) cancer incidence % in OU: Overall = 7.4 (1.8, 9.0); studies with general population = 1.8 (1.4, 9.0); studies with NOU = 8.6 (7.6, 12.8)
